# Supplementary figures and images for: Integrating unsupervised language model with triplet neural networks for protein gene ontology prediction
Source: PLoS Comput Biol. 2022 Dec 22;18(12):e1010793. doi: 10.1371/journal.pcbi.1010793 (PMC9822105; doi:10.1371/journal.pcbi.1010793)

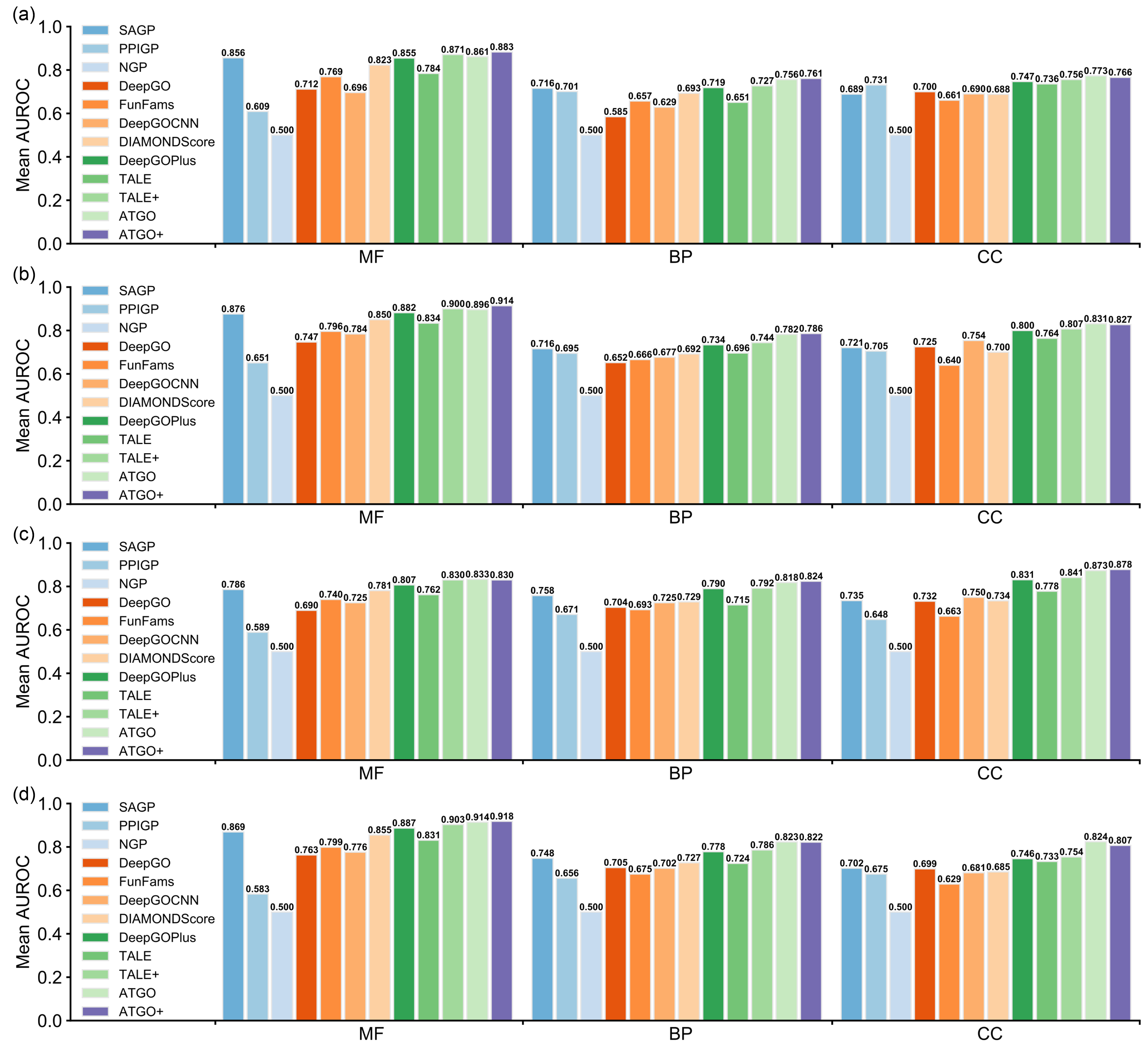

Supplement: S1 Fig — (a) range 5–10. (b) range 10–30. (c) range 30–50. (d) range >50. (TIF) [file pcbi.1010793.s001.tif]

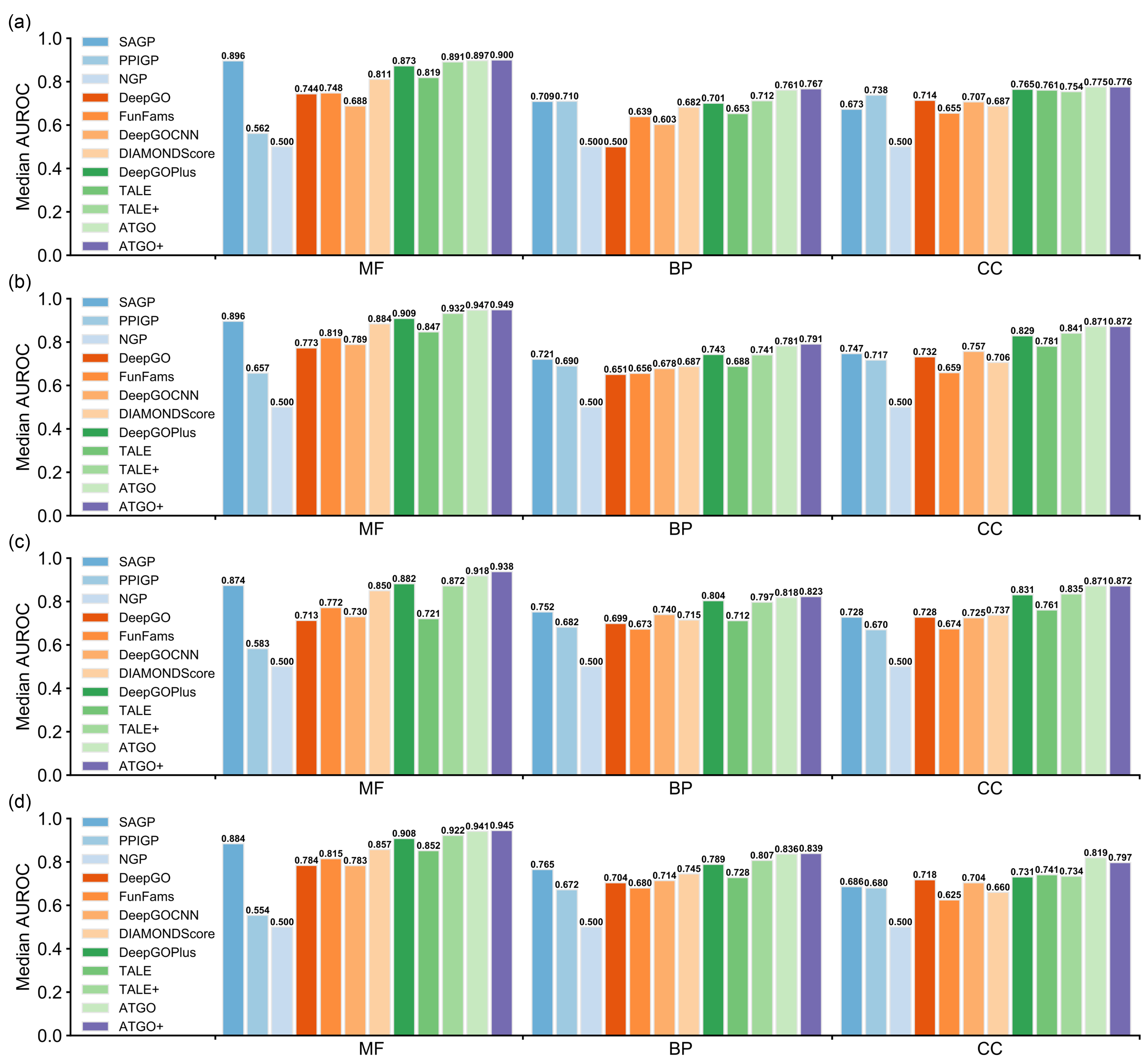

Supplement: S2 Fig — (a) range 5–10. (b) range 10–30. (c) range 30–50. (d) range >50. (TIF) [file pcbi.1010793.s002.tif]

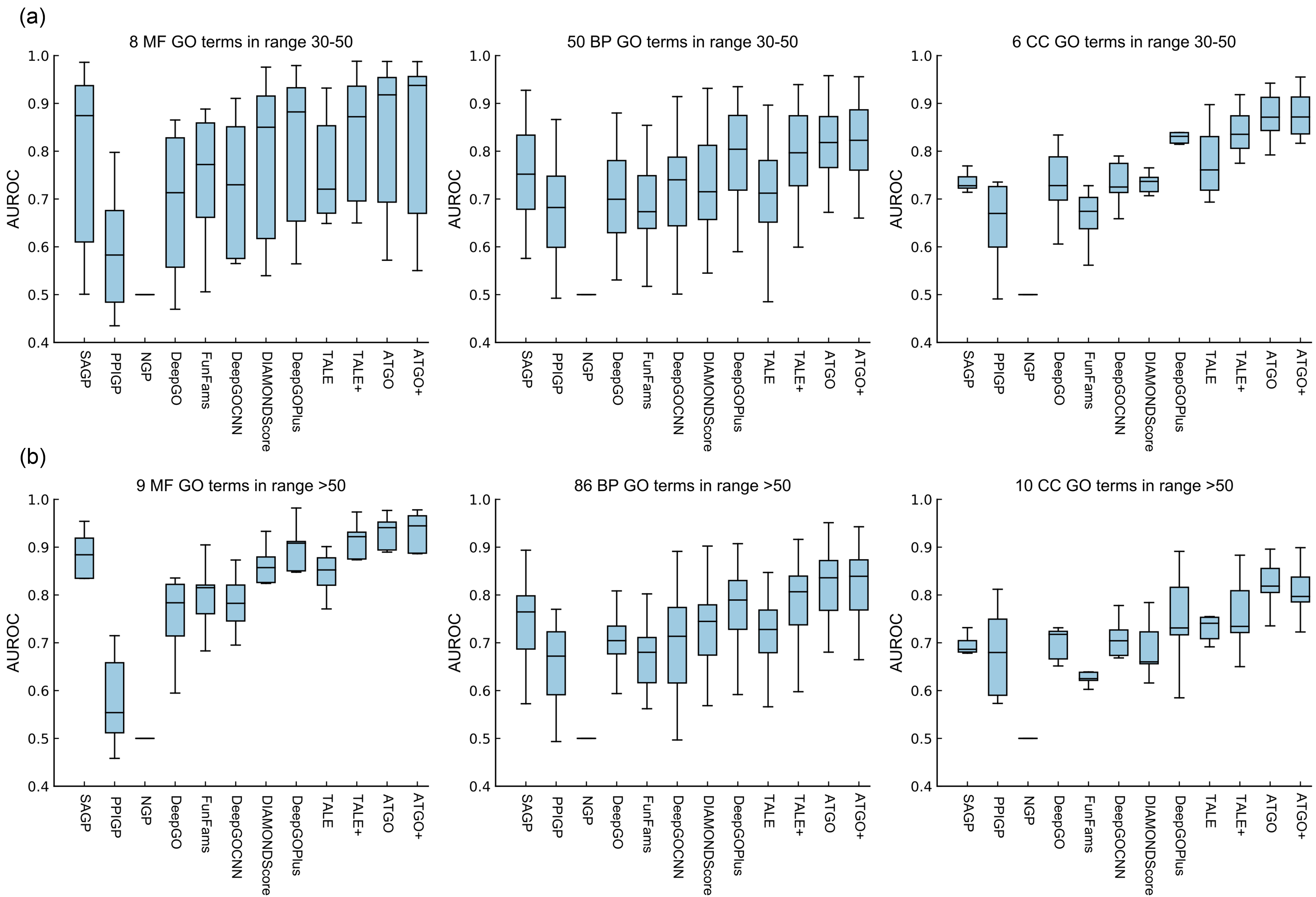

Supplement: S3 Fig — (a) range 30–50. (b) range >50. (TIF) [file pcbi.1010793.s003.tif]

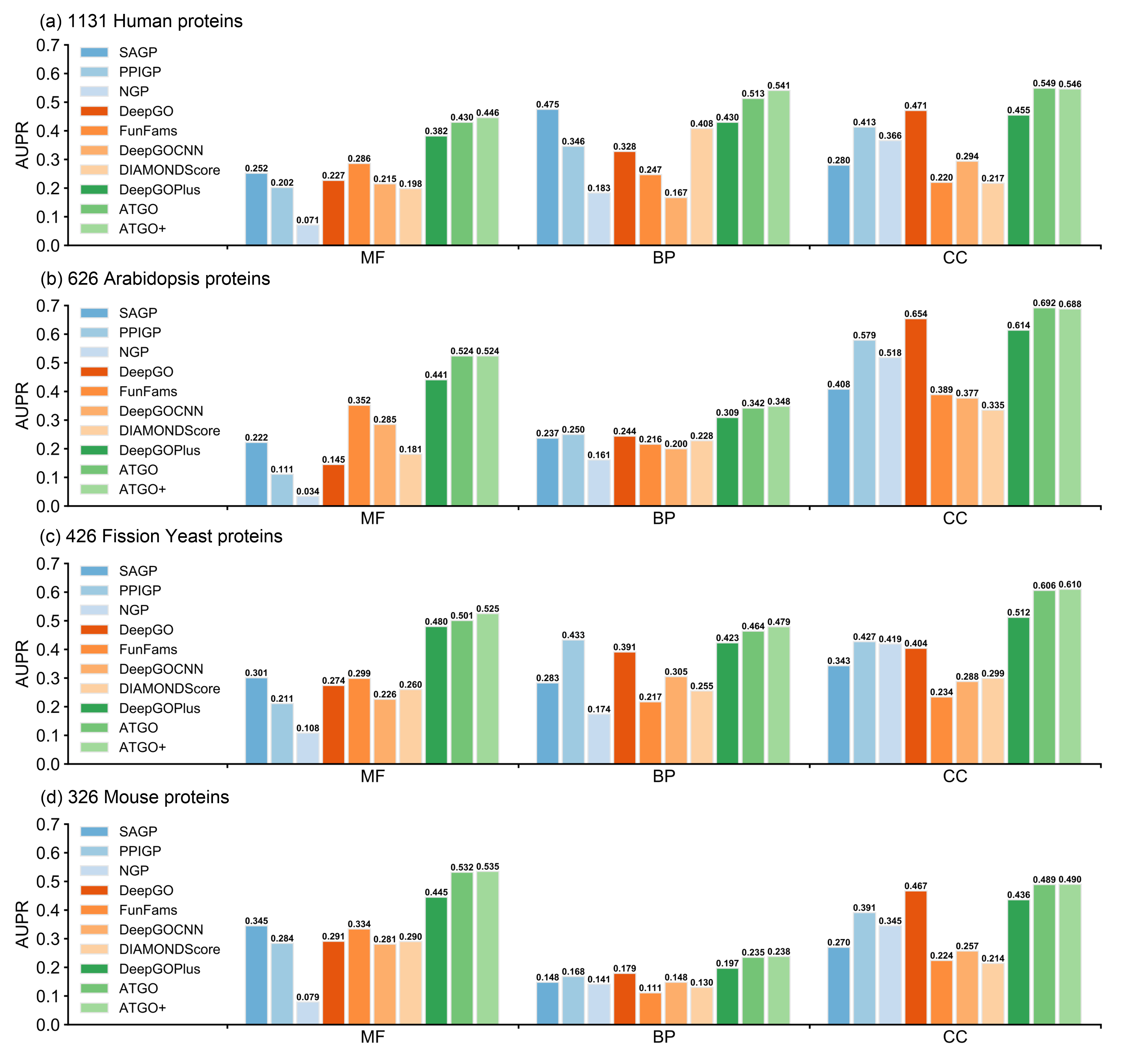

Supplement: S4 Fig — (a) Human (b) Arabidopsis (c) Fission Yeast (d) Mouse. (TIF) [file pcbi.1010793.s004.tif]

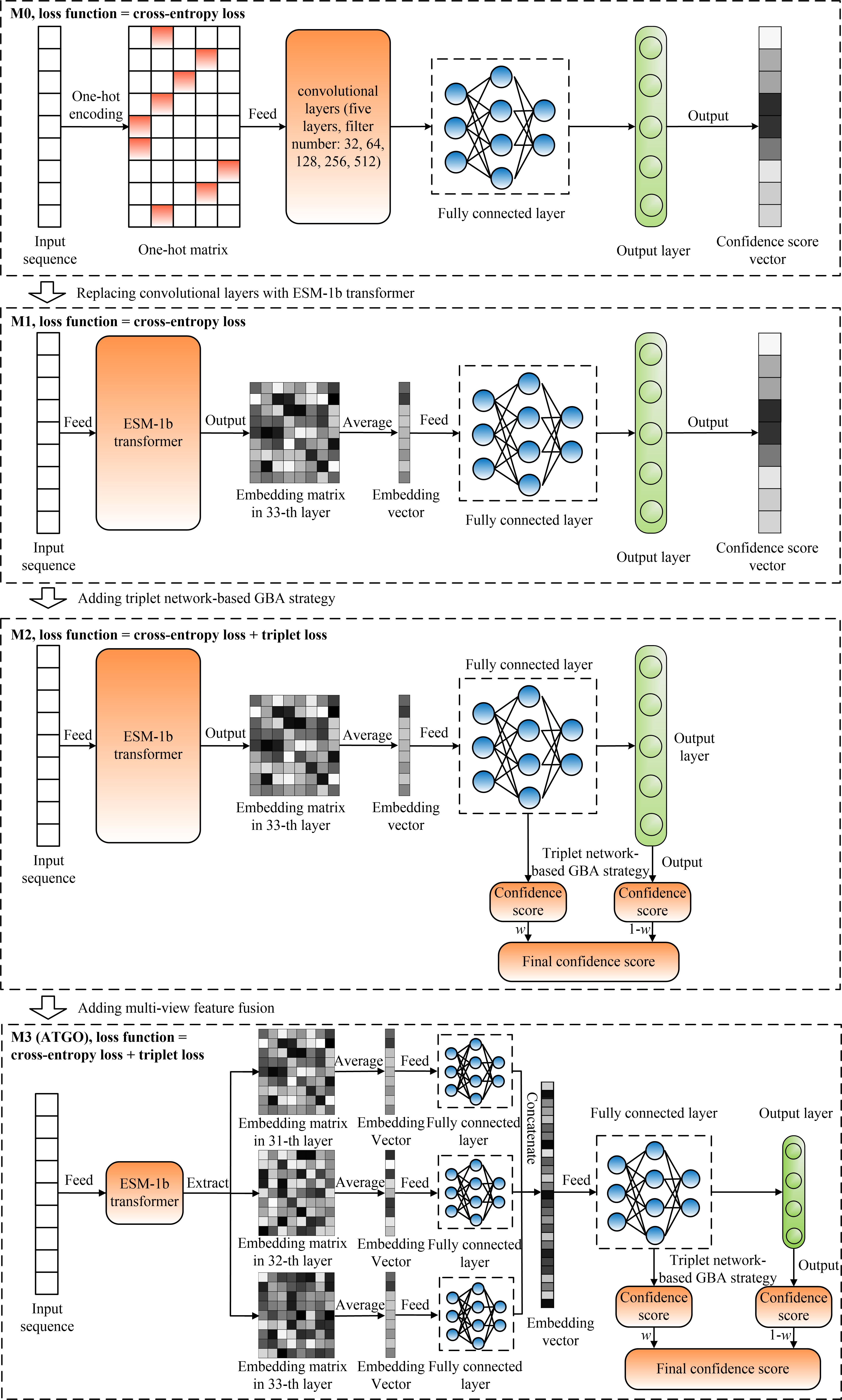

Supplement: S5 Fig — (TIF) [file pcbi.1010793.s005.tif]
