# Supplementary material for: Integrating unsupervised language model with triplet neural networks for protein gene ontology prediction
Source: PLoS Comput Biol. 2022 Dec 22;18(12):e1010793. doi: 10.1371/journal.pcbi.1010793 (PMC9822105; doi:10.1371/journal.pcbi.1010793)
Supplement: S2 Table — (DOCX) [file pcbi.1010793.s007.docx]

**S2 Table**. The statistic values between SAGP and ATGO in Group A under Nemenyi post-hoc test on 1068 test proteins for MF aspect versus the increase of $K$.

| $K$ | 2 | 3 | 4 | 5 | 6 | 7 | 8 | 9 | 10 | 11 | 12 |
| --- | --- | --- | --- | --- | --- | --- | --- | --- | --- | --- | --- |
| $DAR$ | 0.1525 | 0.1802 | 0.2149 | 0.2452 | 0.2539 | 0.2886 | 0.3544 | 0.4133 | 0.4818 | 0.4896 | 0.5641 |
| $\sqrt{K(K+1)/6N}$ | 0.0416 | 0.0589 | 0.0760 | 0.0931 | 0.1101 | 0.1272 | 0.1442 | 0.1612 | 0.1783 | 0.1953 | 0.2123 |
| $q_{\alpha}$ | 3.6635 | 3.0615 | 2.8274 | 2.6344 | 2.3052 | 2.2689 | 2.4576 | 2.5636 | 2.7029 | 2.5074 | 2.6575 |
| *p*-value | 1.0e-03 | 6.2e-03 | 2.4e-02 | 6.4e-02 | 1.9e-01 | 2.6e-01 | 2.1e-01 | 2.0e-01 | 1.7e-01 | 3.0e-01 | 2.5e-01 |
