# Supplementary material for: Integrating unsupervised language model with triplet neural networks for protein gene ontology prediction
Source: PLoS Comput Biol. 2022 Dec 22;18(12):e1010793. doi: 10.1371/journal.pcbi.1010793 (PMC9822105; doi:10.1371/journal.pcbi.1010793)
Supplement: S3 Table — p-values in parenthesis are calculated between ATGO and TALE by two-sided Student’s t-test. Specifically, the proposed ATGO is repeatedly implemented with 10 times on the benchmark dataset to generate the corresponding performance evaluation indices, which are compared with the fixed evaluation index generated by TALE to calculate p-value using two-sided Student’s t-test. Bold fonts highlight the best performer in each category. (DOCX) [file pcbi.1010793.s008.docx]

**S3 Table**. The prediction performance with including root GO terms for ATGO and TALE on all 1068 test proteins. *p*-values in parenthesis are calculated between ATGO and TALE by two-sided Student’s t-test. Specifically, the proposed ATGO is repeatedly implemented with 10 times on the benchmark dataset to generate the corresponding performance evaluation indices, which are compared with the fixed evaluation index generated by TALE to calculate *p*-value using two-sided Student’s t-test. Bold fonts highlight the best performer in each category.

| **Methods** | **F_max_** | | | **AUPR** | | |
| --- | --- | --- | --- | --- | --- | --- |
|  | **MF** | **BP** | **CC** | **MF** | **BP** | **CC** |
| TALE | 0.549  (7.3e-16) | 0.361  (2.4e-16) | 0.600  (1.1e-16) | 0.383  (2.6e-18) | 0.254  (1.4e-18) | 0.438  (4.7e-17) |
| ATGO | **0.688** | **0.465** | **0.695** | **0.689** | **0.413** | **0.654** |
