# Supplementary material for: Integrating unsupervised language model with triplet neural networks for protein gene ontology prediction
Source: PLoS Comput Biol. 2022 Dec 22;18(12):e1010793. doi: 10.1371/journal.pcbi.1010793 (PMC9822105; doi:10.1371/journal.pcbi.1010793)
Supplement: S4 Table — p-values in parenthesis are calculated between ATGO and other single-based methods and between ATGO+ and other composite methods by two-sided Student’s t-test. Specifically, the proposed ATGO and ATGO+ are repeatedly implemented with 10 times on the benchmark dataset to generate the corresponding performance evaluation indices, which are compared with the fixed evaluation index generated by the competing method to calculate p-value using two-sided Student’s t-test. Bold fonts highlight the best performer in each category. (DOCX) [file pcbi.1010793.s009.docx]

**S4 Table.** The summary of the proposed ATGO/ATGO+ and other 10 competing GO prediction methods on a subset of 562 test proteins which have available templates or interaction partners in all of SAGP, PPIGP, FunFams, and DIAMONDScore. *p*-values in parenthesis are calculated between ATGO and other single-based methods and between ATGO+ and other composite methods by two-sided Student’s t-test. Specifically, the proposed ATGO and ATGO+ are repeatedly implemented with 10 times on the benchmark dataset to generate the corresponding performance evaluation indices, which are compared with the fixed evaluation index generated by the competing method to calculate *p*-value using two-sided Student’s t-test. Bold fonts highlight the best performer in each category.

| **Methods** | | **F_max_** | | | **AUPR** | | |
| --- | --- | --- | --- | --- | --- | --- | --- |
|  |  | **MF** | **BP** | **CC** | **MF** | **BP** | **CC** |
| Single algorithms | SAGP | 0.637 | 0.418 | 0.598 | 0.412 | 0.274 | 0.422 |
|  |  | (1.2e-06) | (4.6e-08) | (1.9e-10) | (5.2e-17) | (2.0e-15) | (8.5e-19) |
|  | PPIGP | 0.332 | 0.387 | 0.590 | 0.209 | 0.294 | 0.537 |
|  |  | (2.9e-17) | (1.2e-12) | (4.1e-11) | (1.5e-19) | (1.9e-14) | (1.4e-15) |
|  | NGP | 0.246 | 0.272 | 0.525 | 0.121 | 0.174 | 0.404 |
|  |  | (3.5e-18) | (1.2e-17) | (2.6e-14) | (2.7e-20) | (2.3e-18) | (4.0e-19) |
|  | DeepGO | 0.383 | 0.347 | 0.547 | 0.318 | 0.250 | 0.495 |
|  |  | (1.3e-16) | (3.9e-15) | (1.8e-13) | (2.1e-18) | (2.3e-16) | (4.5e-17) |
|  | FunFams | 0.512 | 0.343 | 0.482 | 0.325 | 0.176 | 0.293 |
|  |  | (3.9e-14) | (2.4e-15) | (1.5e-15) | (2.6e-18) | (2.5e-18) | (1.0e-20) |
|  | DeepGOCNN | 0.352 | 0.309 | 0.494 | 0.282 | 0.212 | 0.366 |
|  |  | (5.2e-17) | (1.4e-16) | (3.1e-15) | (8.1e-19) | (1.7e-17) | (9.6e-20) |
|  | DIAMONDScore | 0.629 | 0.405 | 0.580 | 0.322 | 0.232 | 0.316 |
|  |  | (7.3e-08) | (1.1e-10) | (8.6e-12) | (2.4e-18) | (6.1e-17) | (2.0e-20) |
|  | TALE | 0.397 | 0.318 | 0.527 | 0.338 | 0.243 | 0.499 |
|  |  | (2.2e-16) | (2.7e-16) | (3.0e-14) | (3.9e-18) | (1.3e-16) | (6.0e-17) |
|  | ATGO | 0.662 | 0.439 | 0.645 | 0.647 | 0.373 | 0.633 |
| Composite algorithms | DeepGOPlus | 0.641 | 0.412 | 0.580 | 0.581 | 0.335 | 0.543 |
|  |  | (9.8e-09) | (2.0e-10) | (6.9e-14) | (2.4e-13) | (4.5e-16) | (1.3e-16) |
|  | TALE+ | 0.640 | 0.423 | 0.611 | 0.588 | 0.346 | 0.621 |
|  |  | (7.1e-09) | (1.2e-08) | (1.7e-11) | (6.5e-13) | (6.4e-15) | (5.1e-10) |
|  | ATGO+ | **0.666** | **0.445** | **0.648** | **0.651** | **0.383** | **0.643** |
