# Supplementary material for: Integrating unsupervised language model with triplet neural networks for protein gene ontology prediction
Source: PLoS Comput Biol. 2022 Dec 22;18(12):e1010793. doi: 10.1371/journal.pcbi.1010793 (PMC9822105; doi:10.1371/journal.pcbi.1010793)
Supplement: S5 Table — Bold fonts highlight the best performer in each category. (DOCX) [file pcbi.1010793.s010.docx]

**S5** **Table.** The ICW-F_max_ values of 12 GO prediction methods on the 1068 benchmark proteins. Bold fonts highlight the best performer in each category.

| **Methods** | | **ICW-F_max_** | | |
| --- | --- | --- | --- | --- |
|  |  | **MF** | **BP** | **CC** |
| Single algorithms | SAGP | 0.562 | 0.329 | 0.393 |
|  | PPIGP | 0.199 | 0.240 | 0.350 |
|  | NGP | 0.195 | 0.167 | 0.250 |
|  | DeepGO | 0.315 | 0.232 | 0.324 |
|  | FunFams | 0.435 | 0.255 | 0.322 |
|  | DeepGOCNN | 0.273 | 0.213 | 0.190 |
|  | DIAMONDScore | 0.560 | 0.325 | 0.387 |
|  | TALE | 0.351 | 0.217 | 0.257 |
|  | ATGO | 0.590 | 0.347 | 0.486 |
| Composite algorithms | DeepGOPlus | 0.569 | 0.340 | 0.350 |
|  | TALE+ | 0.569 | 0.350 | 0.439 |
|  | ATGO+ | **0.595** | **0.367** | **0.488** |
