# Supplementary material for: Integrating unsupervised language model with triplet neural networks for protein gene ontology prediction
Source: PLoS Comput Biol. 2022 Dec 22;18(12):e1010793. doi: 10.1371/journal.pcbi.1010793 (PMC9822105; doi:10.1371/journal.pcbi.1010793)
Supplement: S6 Table — Because the p-values can be only approximated in the range from 1.0e-03 to 9.0e-01 under post-hoc Nemenyi test using Python package, the numerical value of 1.0e-03 (or 9.0e-01) means that the p-value is below to 1.0e-03 (or upon to 9.0e-01). (DOCX) [file pcbi.1010793.s011.docx]

**S6 Table**. The *p*-values of performance difference between 10 GO prediction methods on 3328 individual CAFA3 targets under post-hoc Nemenyi test at the individual protein level, where the performance of each prediction method is measured by a group of F_1_-scores, each of which is calculated from the predicted GO terms and native GO annotation in a single test protein. Because the *p*-values can be only approximated in the range from 1.0e-03 to 9.0e-01 under post-hoc Nemenyi test using Python package, the numerical value of 1.0e-03 (or 9.0e-01) means that the *p*-value is below to 1.0e-03 (or upon to 9.0e-01).

|  | Methods | SAGP | PPIGP | NGP | DeepGO | FunFams | DeepGOCNN | DIAMONDScore | ATGO | DeepGOPlus | ATGO+ |
| --- | --- | --- | --- | --- | --- | --- | --- | --- | --- | --- | --- |
| MF | SAGP | 1.0e+00 | 1.0e-03 | 1.0e-03 | 1.0e-03 | 1.0e-03 | 1.8e-03 | 9.0e-01 | 1.0e-03 | 9.0e-01 | 1.0e-03 |
|  | PPIGP | 1.0e-03 | 1.0e+00 | 1.0e-03 | 8.1e-01 | 9.0e-01 | 4.0e-02 | 1.0e-03 | 1.0e-03 | 1.0e-03 | 1.0e-03 |
|  | NGP | 1.0e-03 | 1.0e-03 | 1.0e+00 | 1.0e-03 | 1.0e-03 | 1.0e-03 | 1.0e-03 | 1.0e-03 | 1.0e-03 | 1.0e-03 |
|  | DeepGO | 1.0e-03 | 8.1e-01 | 1.0e-03 | 1.0e+00 | 3.4e-01 | 8.3e-01 | 1.0e-03 | 1.0e-03 | 1.0e-03 | 1.0e-03 |
|  | FunFams | 1.0e-03 | 9.0e-01 | 1.0e-03 | 3.4e-01 | 1.0e+00 | 2.7e-03 | 1.0e-03 | 1.0e-03 | 1.0e-03 | 1.0e-03 |
|  | DeepGOCNN | 1.8e-03 | 4.0e-02 | 1.0e-03 | 8.3e-01 | 2.7e-03 | 1.0e+00 | 1.0e-02 | 1.0e-03 | 1.0e-03 | 1.0e-03 |
|  | DIAMONDScore | 9.0e-01 | 1.0e-03 | 1.0e-03 | 1.0e-03 | 1.0e-03 | 1.0e-02 | 1.0e+00 | 1.0e-03 | 9.0e-01 | 1.0e-03 |
|  | ATGO | 1.0e-03 | 1.0e-03 | 1.0e-03 | 1.0e-03 | 1.0e-03 | 1.0e-03 | 1.0e-03 | 1.0e+00 | 1.0e-03 | 9.0e-01 |
|  | DeepGOPlus | 9.0e-01 | 1.0e-03 | 1.0e-03 | 1.0e-03 | 1.0e-03 | 1.0e-03 | 9.0e-01 | 1.0e-03 | 1.0e+00 | 1.0e-03 |
|  | ATGO+ | 1.0e-03 | 1.0e-03 | 1.0e-03 | 1.0e-03 | 1.0e-03 | 1.0e-03 | 1.0e-03 | 9.0e-01 | 1.0e-03 | 1.0e+00 |
| BP | SAGP | 1.0e+00 | 1.0e-03 | 1.0e-03 | 1.2e-02 | 1.0e-03 | 1.0e-03 | 1.0e-03 | 1.0e-03 | 9.0e-01 | 1.0e-03 |
|  | PPIGP | 1.0e-03 | 1.0e+00 | 7.9e-03 | 1.0e-03 | 1.0e-03 | 1.0e-03 | 8.6e-02 | 1.0e-03 | 1.0e-03 | 1.0e-03 |
|  | NGP | 1.0e-03 | 7.9e-03 | 1.0e+00 | 1.0e-03 | 1.0e-03 | 1.5e-02 | 1.0e-03 | 1.0e-03 | 1.0e-03 | 1.0e-03 |
|  | DeepGO | 1.2e-02 | 1.0e-03 | 1.0e-03 | 1.0e+00 | 1.0e-03 | 1.0e-03 | 4.9e-01 | 1.0e-03 | 4.8e-02 | 1.0e-03 |
|  | FunFams | 1.0e-03 | 1.0e-03 | 1.0e-03 | 1.0e-03 | 1.0e+00 | 1.0e-03 | 1.0e-03 | 1.0e-03 | 1.0e-03 | 1.0e-03 |
|  | DeepGOCNN | 1.0e-03 | 1.0e-03 | 1.5e-02 | 1.0e-03 | 1.0e-03 | 1.0e+00 | 1.0e-03 | 1.0e-03 | 1.0e-03 | 1.0e-03 |
|  | DIAMONDScore | 1.0e-03 | 8.6e-02 | 1.0e-03 | 4.9e-01 | 1.0e-03 | 1.0e-03 | 1.0e+00 | 1.0e-03 | 1.0e-03 | 1.0e-03 |
|  | ATGO | 1.0e-03 | 1.0e-03 | 1.0e-03 | 1.0e-03 | 1.0e-03 | 1.0e-03 | 1.0e-03 | 1.0e+00 | 1.0e-03 | 3.9e-01 |
|  | DeepGOPlus | 9.0e-01 | 1.0e-03 | 1.0e-03 | 4.8e-02 | 1.0e-03 | 1.0e-03 | 1.0e-03 | 1.0e-03 | 1.0e+00 | 1.0e-03 |
|  | ATGO+ | 1.0e-03 | 1.0e-03 | 1.0e-03 | 1.0e-03 | 1.0e-03 | 1.0e-03 | 1.0e-03 | 3.9e-01 | 1.0e-03 | 1.0e+00 |
| CC | SAGP | 1.0e+00 | 9.0e-01 | 9.0e-01 | 1.0e-03 | 1.0e-03 | 1.0e-03 | 2.9e-01 | 1.0e-03 | 8.9e-01 | 1.0e-03 |
|  | PPIGP | 9.0e-01 | 1.0e+00 | 9.0e-01 | 1.0e-03 | 1.0e-03 | 1.0e-03 | 9.0e-01 | 1.0e-03 | 8.5e-02 | 1.0e-03 |
|  | NGP | 9.0e-01 | 9.0e-01 | 1.0e+00 | 1.0e-03 | 1.0e-03 | 1.0e-03 | 9.0e-01 | 1.0e-03 | 8.9e-02 | 1.0e-03 |
|  | DeepGO | 1.0e-03 | 1.0e-03 | 1.0e-03 | 1.0e+00 | 1.0e-03 | 1.0e-03 | 1.0e-03 | 1.0e-03 | 3.8e-03 | 1.0e-03 |
|  | FunFams | 1.0e-03 | 1.0e-03 | 1.0e-03 | 1.0e-03 | 1.0e+00 | 1.8e-01 | 1.0e-03 | 1.0e-03 | 1.0e-03 | 1.0e-03 |
|  | DeepGOCNN | 1.0e-03 | 1.0e-03 | 1.0e-03 | 1.0e-03 | 1.8e-01 | 1.0e+00 | 1.0e-03 | 1.0e-03 | 1.0e-03 | 1.0e-03 |
|  | DIAMONDScore | 2.9e-01 | 9.0e-01 | 9.0e-01 | 1.0e-03 | 1.0e-03 | 1.0e-03 | 1.0e+00 | 1.0e-03 | 3.0e-03 | 1.0e-03 |
|  | ATGO | 1.0e-03 | 1.0e-03 | 1.0e-03 | 1.0e-03 | 1.0e-03 | 1.0e-03 | 1.0e-03 | 1.0e+00 | 1.0e-03 | 7.4e-01 |
|  | DeepGOPlus | 8.9e-01 | 8.5e-02 | 8.9e-02 | 3.8e-03 | 1.0e-03 | 1.0e-03 | 3.0e-03 | 1.0e-03 | 1.0e+00 | 1.0e-03 |
|  | ATGO+ | 1.0e-03 | 1.0e-03 | 1.0e-03 | 1.0e-03 | 1.0e-03 | 1.0e-03 | 1.0e-03 | 7.4e-01 | 1.0e-03 | 1.0e+00 |
