# Supplementary material for: Integrating unsupervised language model with triplet neural networks for protein gene ontology prediction
Source: PLoS Comput Biol. 2022 Dec 22;18(12):e1010793. doi: 10.1371/journal.pcbi.1010793 (PMC9822105; doi:10.1371/journal.pcbi.1010793)
Supplement: S7 Table — Bold fonts highlight the best performer in each category. (DOCX) [file pcbi.1010793.s012.docx]

**S7 Table.** The ICW-F_max_ values of 10 GO prediction methods on 3328 CAFA3 targets where a sequence identity cut-off $t_{1}=30\%$ between the training and testing proteins was applied to the five in-house methods (ATGO, ATGO+, SAGP, PPIGP, and NGP). Bold fonts highlight the best performer in each category.

| **Methods** | | **ICW-F_max_** | | |
| --- | --- | --- | --- | --- |
|  |  | **MF** | **BP** | **CC** |
| Single algorithms | SAGP | 0.439 | 0.413 | 0.355 |
|  | PPIGP | 0.226 | 0.303 | 0.343 |
|  | NGP | 0.143 | 0.175 | 0.314 |
|  | DeepGO | 0.256 | 0.279 | 0.357 |
|  | FunFams | 0.455 | 0.375 | 0.391 |
|  | DeepGOCNN | 0.291 | 0.211 | 0.163 |
|  | DIAMONDScore | 0.431 | 0.391 | 0.356 |
|  | ATGO | 0.479 | 0.399 | 0.426 |
| Composite algorithms | DeepGOPlus | 0.438 | 0.389 | 0.353 |
|  | ATGO+ | **0.487** | **0.437** | **0.426** |
