# Supplementary material for: Integrating unsupervised language model with triplet neural networks for protein gene ontology prediction
Source: PLoS Comput Biol. 2022 Dec 22;18(12):e1010793. doi: 10.1371/journal.pcbi.1010793 (PMC9822105; doi:10.1371/journal.pcbi.1010793)
Supplement: S8 Table — Bold fonts highlight the best performer in each category. (DOCX) [file pcbi.1010793.s013.docx]

**S8 Table.** The performance of 10 GO prediction methods under the cut-off $t_{1}=30\%$ on 1177 no-knowledge (NK) and 2151 limited-knowledge (LK) CAFA3 proteins. Bold fonts highlight the best performer in each category.

| **Datasets** | **Methods** | **F_max_** | | | **AUPR** | | | **Coverage** | | |
| --- | --- | --- | --- | --- | --- | --- | --- | --- | --- | --- |
|  |  | **MF** | **BP** | **CC** | **MF** | **BP** | **CC** | **MF** | **BP** | **CC** |
| NK proteins | SAGP | 0.467 | 0.351 | 0.472 | 0.248 | 0.189 | 0.280 | 0.84 | 0.85 | 0.83 |
|  | PPIGP | 0.286 | 0.316 | 0.476 | 0.176 | 0.212 | 0.440 | 0.85 | 0.83 | 0.84 |
|  | NGP | 0.184 | 0.260 | 0.467 | 0.083 | 0.159 | 0.380 | 1.00 | 1.00 | 1.00 |
|  | DeepGO | 0.302 | 0.332 | 0.502 | 0.230 | 0.233 | 0.501 | 1.00 | 1.00 | 1.00 |
|  | FunFams | 0.461 | 0.356 | 0.430 | 0.282 | 0.181 | 0.250 | 0.63 | 0.63 | 0.61 |
|  | DeepGOCNN | 0.267 | 0.304 | 0.428 | 0.203 | 0.193 | 0.297 | 1.00 | 1.00 | 1.00 |
|  | DIAMONDScore | 0.463 | 0.350 | 0.462 | 0.196 | 0.171 | 0.225 | 0.78 | 0.79 | 0.78 |
|  | ATGO | 0.513 | 0.393 | 0.557 | 0.472 | 0.314 | **0.559** | 1.00 | 1.00 | 1.00 |
|  | DeepGOPlus | 0.473 | 0.373 | 0.473 | 0.385 | 0.269 | 0.472 | 1.00 | 1.00 | 1.00 |
|  | ATGO+ | **0.523** | **0.396** | **0.557** | **0.482** | **0.316** | 0.555 | 1.00 | 1.00 | 1.00 |
| LK proteins | SAGP | 0.461 | 0.548 | 0.479 | 0.241 | 0.392 | 0.322 | 0.81 | 0.93 | 0.88 |
|  | PPIGP | 0.224 | 0.422 | 0.421 | 0.138 | 0.357 | 0.394 | 0.92 | 0.92 | 0.83 |
|  | NGP | 0.142 | 0.339 | 0.416 | 0.055 | 0.175 | 0.348 | 1.00 | 1.00 | 1.00 |
|  | DeepGO | 0.259 | 0.423 | 0.469 | 0.176 | 0.333 | 0.468 | 1.00 | 1.00 | 1.00 |
|  | FunFams | 0.481 | 0.472 | 0.508 | 0.320 | 0.243 | 0.332 | 0.67 | 0.76 | 0.75 |
|  | DeepGOCNN | 0.342 | 0.284 | 0.392 | 0.251 | 0.191 | 0.275 | 1.00 | 1.00 | 1.00 |
|  | DIAMONDScore | 0.452 | 0.518 | 0.469 | 0.200 | 0.344 | 0.256 | 0.74 | 0.89 | 0.84 |
|  | ATGO | 0.498 | 0.564 | 0.523 | 0.468 | 0.465 | 0.528 | 1.00 | 1.00 | 1.00 |
|  | DeepGOPlus | 0.449 | 0.524 | 0.478 | 0.394 | 0.401 | 0.470 | 1.00 | 1.00 | 1.00 |
|  | ATGO+ | **0.511** | **0.574** | **0.525** | **0.473** | **0.488** | **0.534** | 1.00 | 1.00 | 1.00 |
