# Supplementary material for: Integrating unsupervised language model with triplet neural networks for protein gene ontology prediction
Source: PLoS Comput Biol. 2022 Dec 22;18(12):e1010793. doi: 10.1371/journal.pcbi.1010793 (PMC9822105; doi:10.1371/journal.pcbi.1010793)
Supplement: S9 Table — (DOCX) [file pcbi.1010793.s014.docx]

**S9** **Table.** The numbers of proteins for 20 species in CAFA3 test dataset.

| **Species name** | **Taxonomy ID** | **Sample number** |
| --- | --- | --- |
| Human | 9606 | 1131 |
| Arabidopsis | 3702 | 626 |
| Fission Yeast | 284812 | 426 |
| Mouse | 10090 | 326 |
| Escherichia Coli | 83333 | 224 |
| Fly | 7227 | 209 |
| Rat | 10116 | 97 |
| Bacillus Subtilis | 224308 | 76 |
| Dictyostelium Discoideum | 44689 | 49 |
| Zebrafish | 7955 | 46 |
| Budding Yeast | 559292 | 32 |
| Candida Albicans | 237561 | 27 |
| Salmonella Enterica | 99287 | 16 |
| Xenopus Laevis | 8355 | 14 |
| Methanocaldococcus Jannaschii | 243232 | 7 |
| Pseudomonas Putida | 160488 | 7 |
| Helicobacter Pylori | 85962 | 5 |
| Saccharolobus Solfataricus P2 | 273057 | 4 |
| Mycoplasma Genitalium | 243273 | 3 |
| Pseudomonas Aeruginosa | 208963 | 3 |
