# Supplementary material for: Integrating unsupervised language model with triplet neural networks for protein gene ontology prediction
Source: PLoS Comput Biol. 2022 Dec 22;18(12):e1010793. doi: 10.1371/journal.pcbi.1010793 (PMC9822105; doi:10.1371/journal.pcbi.1010793)
Supplement: S10 Table — Bold fonts highlight the best performer in each category. (DOCX) [file pcbi.1010793.s015.docx]

**S10 Table**. The prediction performance of 5 GO prediction methods under the cut-off $t_{1}=100\%$ on CAFA3 test proteins. Bold fonts highlight the best performer in each category.

| **Datasets** | **Methods** | **F_max_** | | | **AUPR** | | |
| --- | --- | --- | --- | --- | --- | --- | --- |
|  |  | **MF** | **BP** | **CC** | **MF** | **BP** | **CC** |
| All 3328 proteins | SAGP | 0.520 | 0.515 | 0.504 | 0.328 | 0.366 | 0.350 |
|  | PPIGP | 0.253 | 0.390 | 0.473 | 0.160 | 0.312 | 0.461 |
|  | NGP | 0.166 | 0.302 | 0.445 | 0.065 | 0.170 | 0.366 |
|  | ATGO | 0.548 | 0.520 | 0.555 | 0.504 | 0.445 | 0.551 |
|  | ATGO+ | **0.551** | **0.540** | **0.559** | **0.514** | **0.470** | **0.546** |
| 1177 no-knowledge proteins | SAGP | 0.494 | 0.387 | 0.509 | 0.297 | 0.230 | 0.337 |
|  | PPIGP | 0.299 | 0.335 | 0.491 | 0.189 | 0.236 | 0.471 |
|  | NGP | 0.192 | 0.260 | 0.467 | 0.082 | 0.160 | 0.380 |
|  | ATGO | **0.533** | 0.400 | 0.569 | 0.476 | 0.338 | 0.562 |
|  | ATGO+ | 0.532 | **0.419** | **0.570** | **0.490** | **0.346** | **0.553** |
| 2151 limited-knowledge proteins | SAGP | 0.537 | 0.602 | 0.498 | 0.347 | 0.473 | 0.366 |
|  | PPIGP | 0.221 | 0.435 | 0.456 | 0.140 | 0.366 | 0.447 |
|  | NGP | 0.147 | 0.339 | 0.416 | 0.055 | 0.175 | 0.348 |
|  | ATGO | 0.562 | 0.602 | 0.539 | 0.526 | 0.529 | 0.535 |
|  | ATGO+ | **0.566** | **0.622** | **0.542** | **0.532** | **0.564** | **0.537** |
