# Supplementary material for: Integrating unsupervised language model with triplet neural networks for protein gene ontology prediction
Source: PLoS Comput Biol. 2022 Dec 22;18(12):e1010793. doi: 10.1371/journal.pcbi.1010793 (PMC9822105; doi:10.1371/journal.pcbi.1010793)
Supplement: S11 Table — Bold fonts highlight the best performer in each category. (DOCX) [file pcbi.1010793.s016.docx]

**S11 Table.** The prediction performance of SAGP and BLAST baseline on our constructed test dataset and CAFA3 test dataset with different cut-off values of sequence identity**.** Bold fonts highlight the best performer in each category.

| **Datasets** | **Methods** | **F_max_** | | | **AUPR** | | |
| --- | --- | --- | --- | --- | --- | --- | --- |
|  |  | **MF** | **BP** | **CC** | **MF** | **BP** | **CC** |
| 1068 test proteins  constructed in this work | BLAST baseline | 0.440 | 0.292 | 0.375 | 0.315 | 0.166 | 0.269 |
|  | SAGP | **0.597** | **0.400** | **0.534** | **0.351** | **0.242** | **0.322** |
| 3328 CAFA3 targets  under the cut-off $t_{1}=30\%$ | BLAST baseline | 0.352 | 0.248 | 0.325 | 0.204 | 0.128 | 0.210 |
|  | SAGP | **0.463** | **0.465** | **0.473** | **0.244** | **0.302** | **0.298** |
| 3328 CAFA3 targets  under the cut-off $t_{1}=100\%$ | BLAST baseline | 0.388 | 0.322 | 0.391 | 0.256 | 0.198 | 0.259 |
|  | SAGP | **0.520** | **0.515** | **0.504** | **0.328** | **0.366** | **0.350** |
