# Supplementary material for: Integrating unsupervised language model with triplet neural networks for protein gene ontology prediction
Source: PLoS Comput Biol. 2022 Dec 22;18(12):e1010793. doi: 10.1371/journal.pcbi.1010793 (PMC9822105; doi:10.1371/journal.pcbi.1010793)
Supplement: S12 Table — Bold fonts highlight the best performer in each category. (DOCX) [file pcbi.1010793.s017.docx]

**S12 Table.** The prediction performance of ATGO models via four metric learning methods on two test datasets. Bold fonts highlight the best performer in each category.

| **Datasets** | **Methods** | **F_max_** | | | **AUPR** | | |
| --- | --- | --- | --- | --- | --- | --- | --- |
|  |  | **MF** | **BP** | **CC** | **MF** | **BP** | **CC** |
| 1068 test proteins constructed in this work | F_1_ | 0.627 | 0.425 | 0.623 | 0.603 | 0.361 | **0.600** |
|  | JS | **0.629** | 0.423 | 0.622 | 0.600 | 0.355 | 0.557 |
|  | WF_1_ | 0.628 | **0.426** | 0.623 | **0.606** | **0.364** | 0.579 |
|  | WJS | 0.628 | **0.426** | **0.624** | 0.587 | 0.358 | 0.592 |
| 3328 CAFA3 targets under the cut-off  $t_{1}=30\%$ | F_1_ | **0.501** | 0.495 | 0.542 | **0.469** | 0.397 | 0.546 |
|  | JS | 0.498 | 0.491 | 0.544 | 0.441 | 0.401 | 0.543 |
|  | WF_1_ | 0.500 | 0.492 | **0.545** | 0.429 | 0.404 | **0.550** |
|  | WJS | 0.497 | **0.497** | 0.544 | 0.429 | **0.410** | 0.549 |
