# Supplementary material for: Integrating unsupervised language model with triplet neural networks for protein gene ontology prediction
Source: PLoS Comput Biol. 2022 Dec 22;18(12):e1010793. doi: 10.1371/journal.pcbi.1010793 (PMC9822105; doi:10.1371/journal.pcbi.1010793)
Supplement: S13 Table — (DOCX) [file pcbi.1010793.s018.docx]

**S13 Table.** The incorrectly predicted GO terms for 12 methods on three proteins in BP aspect.

| **Methods** | **A6XMY0** | **E7CIP7** | **F4I082** |
| --- | --- | --- | --- |
| SAGP |  | GO:0044419 GO:0009607 GO:0009605 GO:0043207 GO:0050896 GO:0009617 GO:0006952 GO:0006950 GO:0051707 | GO:0032502 GO:0042335 GO:0006869 GO:0006810 GO:0071702 GO:0051234 GO:0051179 GO:0048856 |
| PPIGP |  |  | GO:0032502 GO:0009628 GO:0044238 GO:0009987 GO:0044237 GO:0071704 GO:0009058 GO:0006807 GO:0065007 GO:0016043 GO:0008152 GO:1901576 GO:0042221 GO:0044249 GO:0050789 GO:0071840 |
| NGP | GO:0032502 GO:0044238 GO:0019222 GO:0060255 GO:0048856 GO:0044237 GO:0050794 GO:0071704 GO:0006807 GO:0065007 GO:0016043 GO:0008152 GO:0048518 GO:0043170 GO:0050789 GO:0071840 | GO:0032502 GO:0048856 GO:0019222 GO:0060255 GO:0050896 GO:0009987 GO:0044237 GO:0050794 GO:0006807 GO:0065007 GO:0016043 GO:0048518 GO:0050789 GO:0071840 | GO:0032502 GO:0044238 GO:0048856 GO:0019222 GO:0060255 GO:0009987 GO:0044237 GO:0050794 GO:0071704 GO:0006807 GO:0065007 GO:0016043 GO:0008152 GO:0048518 GO:0043170 GO:0071840 GO:0050789 |
| DeepGO | GO:0080090 GO:0019222 GO:0031326 GO:0031323 GO:0050789 GO:0071704 GO:2000112 GO:0060255 GO:0065007 GO:0048518 GO:0048519 GO:0065008 GO:0010468 GO:0019219 GO:0048583 GO:0009889 GO:1903506 GO:0050794 GO:0051171 GO:0008152 GO:2001141 GO:0051704 GO:0044238 GO:0051252 GO:0044237 GO:0051239 GO:0010556 GO:0048523 GO:0048522 | GO:2001141 GO:0009987 GO:0080090 GO:0019222 GO:0019219 GO:0009889 GO:0050896 GO:0051252 GO:0031323 GO:1903506 GO:0050794 GO:0006355 GO:0010556 GO:0065007 GO:0051171 GO:0031326 GO:0060255 GO:0010468 GO:2000112 GO:0050789 | GO:0048856 GO:0009892 GO:0080090 GO:0019222 GO:0009891 GO:0031327 GO:0031326 GO:0031325 GO:2000241 GO:0031323 GO:0048580 GO:0010629 GO:0032501 GO:0007165 GO:0050789 GO:0009893 GO:0009755 GO:0010605 GO:0009890 GO:0033993 GO:0031328 GO:2000112 GO:2000113 GO:0009737 GO:0060255 GO:0065007 GO:0048518 GO:0048519 GO:0010468 GO:0032502 GO:0009719 GO:0031324 GO:0048608 GO:0050793 GO:0009987 GO:0009889 GO:1903506 GO:0050794 GO:0003006 GO:0001101 GO:0051239 GO:2001141 GO:0042221 GO:0010033 GO:1901700 GO:0022414 GO:0009725 GO:0051252 GO:0019219 GO:0006355 GO:0010556 GO:2000026 GO:0048522 GO:0097305 GO:0010558 GO:0048523 GO:0051171 |
| FunFams |  | GO:0002376 GO:0009607 GO:0009605 GO:0043207 GO:0050896 GO:0009617 GO:0006952 GO:0006950 GO:0098542 GO:0042742 GO:0044419 GO:0050829 GO:0051707 GO:0006955 |  |
| DeepGOCNN | GO:0048583 GO:0023052 GO:0007165 GO:0023051 GO:0050829 GO:0010646 GO:0050789 GO:0051716 GO:0009966 GO:0051179 GO:0065007 GO:0065009 GO:0003008 GO:0007186 GO:0032502 GO:0032501 GO:0006810 GO:0050794 GO:0050830 GO:0007267 GO:0007154 GO:0051234 GO:0055085 GO:0051704 GO:0010469 GO:0048856 | GO:0032501 GO:0009605 GO:0050896 GO:0009987 GO:0071554 GO:0006950 GO:0065007 GO:0051179 GO:0051704 | GO:0044238 GO:0009987 GO:0044237 GO:0050794 GO:0071704 GO:0065007 GO:0016043 GO:0008152 GO:0051179 GO:0050789 GO:0071840 |
| DIAMONDScore |  | GO:0044419 GO:0009607 GO:0009605 GO:0043207 GO:0050896 GO:0009617 GO:0006952 GO:0006950 GO:0051707 |  |
| TALE | GO:0050829 GO:0032501 GO:0065007 GO:0050789 GO:0050794 | GO:0071554 GO:0009987 | GO:0044238 GO:0009987 GO:0044237 GO:0050794 GO:0071704 GO:0006807 GO:0065007 GO:0008152 GO:0050789 |
| ATGO |  | GO:0044419 GO:0009987 | GO:0032502 GO:0009628 GO:0003006 GO:0009987 GO:0022414 GO:0065007 GO:0050789 |
| DeepGOPlus | GO:0032501 GO:0065007 | GO:0044419 GO:0009617 GO:0009607 GO:0009605 GO:0043207 GO:0050896 GO:0009987 GO:0006952 GO:0006950 GO:0051707 | GO:0009987 |
| TALE+ |  | GO:0009617 GO:0009607 GO:0009605 GO:0043207 GO:0050896 GO:0009987 GO:0044419 GO:0006950 GO:0051707 | GO:0009987 |
| ATGO+ |  | GO:0044419 GO:0050896 | GO:0032502 GO:0042335 GO:0048856 |
