# Supplementary material for: Integrating unsupervised language model with triplet neural networks for protein gene ontology prediction
Source: PLoS Comput Biol. 2022 Dec 22;18(12):e1010793. doi: 10.1371/journal.pcbi.1010793 (PMC9822105; doi:10.1371/journal.pcbi.1010793)
Supplement: S14 Table — (DOCX) [file pcbi.1010793.s019.docx]

**S14** **Table.** The numbers of proteins and GO terms in benchmark dataset.

| **Benchmark dataset** | $\boldsymbol{N}_{\boldsymbol{MF}}^{\boldsymbol{P}}$ | $\boldsymbol{N}_{\boldsymbol{BP}}^{\boldsymbol{P}}$ | $\boldsymbol{N}_{\boldsymbol{CC}}^{\boldsymbol{P}}$ | $\boldsymbol{N}_{\boldsymbol{ALL}}^{\boldsymbol{P}}$ | $\boldsymbol{N}_{\boldsymbol{MF}}^{\boldsymbol{T}}$ | $\boldsymbol{N}_{\boldsymbol{BP}}^{\boldsymbol{T}}$ | $\boldsymbol{N}_{\boldsymbol{CC}}^{\boldsymbol{T}}$ | $\boldsymbol{N}_{\boldsymbol{ALL}}^{\boldsymbol{T}}$ |
| --- | --- | --- | --- | --- | --- | --- | --- | --- |
| Training dataset | 49135 | 79491 | 71982 | 109132 | 6581 | 20882 | 2782 | 30245 |
| Validation dataset | 515 | 860 | 664 | 1089 | 818 | 3894 | 417 | 5129 |
| Test dataset | 577 | 839 | 586 | 1068 | 876 | 3469 | 382 | 4727 |

$N_{MF}^{P}$/$N_{BP}^{P}$/$N_{CC}^{P}$/$N_{ALL}^{P}$: The number of proteins for MF/BP/CC/all three aspects.

$N_{MF}^{T}$/$N_{BP}^{T}$/$N_{CC}^{T}$/$N_{ALL}^{T}$: The number of GO terms for MF/BP/CC/all three aspects.
