# Supplementary material for: Integrating unsupervised language model with triplet neural networks for protein gene ontology prediction
Source: PLoS Comput Biol. 2022 Dec 22;18(12):e1010793. doi: 10.1371/journal.pcbi.1010793 (PMC9822105; doi:10.1371/journal.pcbi.1010793)
Supplement: S15 Table — (DOCX) [file pcbi.1010793.s020.docx]

**S15 Table.** The values of $margin$, $c_{f}$, $\alpha$, and $K$ for three GO aspects

| **GO aspect** | $\boldsymbol{margin}$ | $\boldsymbol{c}_{\boldsymbol{f}}$ | $\boldsymbol{\alpha}$ | $\boldsymbol{K}$ |
| --- | --- | --- | --- | --- |
| MF | 0.1 | 0.8 | 5 | 30 |
| BP | 0.1 | 0.8 | 5 | 100 |
| CC | 0.1 | 0.8 | 5 | 100 |
