# Supplementary material for: Integrating unsupervised language model with triplet neural networks for protein gene ontology prediction
Source: PLoS Comput Biol. 2022 Dec 22;18(12):e1010793. doi: 10.1371/journal.pcbi.1010793 (PMC9822105; doi:10.1371/journal.pcbi.1010793)
Supplement: S1 Text — (DOCX) [file pcbi.1010793.s021.docx]

**S1 Text. Sequence alignment-based GO prediction (SAGP)**

In SAGP, we select the function templates, which share high sequence similarity with the query, to annotate its function. Specifically, for a query sequence, BLAST software [1] is used to scan the corresponding templates with an e-value cutoff of 0.1. The confidence score of the GO term $q$ by SAGP is calculated by

${S\left( q \right)}_{SAGP}=\frac{\sum_{k=1}^{n} b_{k}\cdot I_{k}(q)}{\sum_{k=1}^{n} b_{k}}$ (S1)

where $n$ is the number of templates identified, $b_{k}$ is the bit-score of $k$-th template by BLAST; $I_{k}\left( q \right)=1$, if the $k$-th template is associated with $q$ in the experimental function annotation; otherwise, $I_{k}\left( q \right)=0$.

**Reference**

1. Altschul SF, Madden TL, Schäffer AA, Zhang J, Zhang Z, et al. Gapped BLAST and PSI-BLAST: a new generation of protein database search programs. Nucleic acids research. 1997; 25:3389-402.
