# Supplementary material for: Integrating unsupervised language model with triplet neural networks for protein gene ontology prediction
Source: PLoS Comput Biol. 2022 Dec 22;18(12):e1010793. doi: 10.1371/journal.pcbi.1010793 (PMC9822105; doi:10.1371/journal.pcbi.1010793)
Supplement: S2 Text — (DOCX) [file pcbi.1010793.s022.docx]

**S2 Text. Protein-protein interaction-based GO prediction (PPIGP)**

For a query, we search its interaction partners from the STRING database [1] for functional annotation. Then, we remove the interaction partners which are not found in the training dataset. Finally, the remaining partners are used to annotate the query. The confidence score is calculated using the same scoring function as in SAGP (i.e., Eq. S1 in S1 Text), where $b_{k}$ is the score assigned by STRING as confidence of interaction between the query and the $k$-th partner.

**Reference**

1. Mering Cv, Huynen M, Jaeggi D, Schmidt S, Bork P, et al. STRING: a database of predicted functional associations between proteins. Nucleic acids research. 2003; 31:258-61.
