# Supplementary material for: Integrating unsupervised language model with triplet neural networks for protein gene ontology prediction
Source: PLoS Comput Biol. 2022 Dec 22;18(12):e1010793. doi: 10.1371/journal.pcbi.1010793 (PMC9822105; doi:10.1371/journal.pcbi.1010793)
Supplement: S3 Text — (DOCX) [file pcbi.1010793.s023.docx]

**S3 Text. Naïve-based GO prediction (NGP)**

In NGP, the confidence score that a query is associated with GO term $q$ is calculated by the frequency of $q$ in the training dataset:

${S\left( q \right)}_{NGP}=N(q)/N_{GO}$ (S2)

where $N(q)$ is the number of proteins associated with $q$, and $N_{GO}$ is the number of proteins with at least one annotation for the same GO aspect as $q$. This predictor can be thought of as a prior arising from the overall abundance of a particular annotation in the training dataset.
