# Supplementary material for: Integrating unsupervised language model with triplet neural networks for protein gene ontology prediction
Source: PLoS Comput Biol. 2022 Dec 22;18(12):e1010793. doi: 10.1371/journal.pcbi.1010793 (PMC9822105; doi:10.1371/journal.pcbi.1010793)
Supplement: S4 Text — (DOCX) [file pcbi.1010793.s024.docx]

**S4 Text. Friedman** **and Nemenyi post-hoc tests at the individual protein level**

We use Friedman test [1], one of the most used approaches in analysis of variance, to identify whether there is a significant performance difference among a group of GO prediction methods. If the significance factor (i.e., *p*-value) is below to 0.05 in Friedman test, the Nemenyi post-hoc test [2] is further performed to identify the performance difference between pairwise prediction methods.

It is noted that most of competing methods, such as SAGP, PPIGP, FunFams, and DIAMONDScore, are performed with constant evaluation indices (i.e., F_max_ and AUPR values) in the entire dataset. Therefore, there is no proper statistical test to identify the performance difference between the above-mentioned methods in the entire dataset. In view of this, we perform Friedman and Nemenyi post-hoc tests at the individual protein level rather than the entire dataset level. Specifically, the performance of each prediction method is measured by a set of F_1_-scores, each of which is calculated from the predicted GO terms and native GO annotation in a single protein (see Eq. S32 in Text S10). Moreover, the predicted GO terms of different methods are determined by their own cut-off setting to achieve the highest F_max_ value. Finally, the Friedman and Nemenyi post-hoc tests are performed on the F_1_-scores of individual test proteins to calculate the *p*-values of performance difference among prediction methods.

**Reference**

1. Sheldon MR, Fillyaw MJ, Thompson WD. The use and interpretation of the Friedman test in the analysis of ordinal‐scale data in repeated measures designs. Physiotherapy Research International. 1996; 1:221-8.

2. Hilton A, Armstrong RA. Statnote 6: post-hoc ANOVA tests. Microbiologist. 2006; 1:34-6.
