# Supplementary material for: Integrating unsupervised language model with triplet neural networks for protein gene ontology prediction
Source: PLoS Comput Biol. 2022 Dec 22;18(12):e1010793. doi: 10.1371/journal.pcbi.1010793 (PMC9822105; doi:10.1371/journal.pcbi.1010793)
Supplement: S5 Text — (DOCX) [file pcbi.1010793.s025.docx]

**S5 Text. An explanation for the difference of *p*-value calculations between Student t-test and Nemenyi post-hoc test.**

It can be found that there exists a big gap of *p*-values between Student t-test at *the entire dataset level* and Nemenyi post-hoc test at *the individual protein level*. To explain this observation, we firstly introduce the procedures of the above-mentioned two statistical tests.

**A. Student t-test.** We select one-sample t-test [1] to calculate the *p*-values between ATGO and other 10 competing methods, because each competing method is performed with constant evaluation indices (i.e., F_max_ and AUPR values) in the entire dataset. Specifically, ATGO is repeatedly implemented on the benchmark dataset in $N$ times to generate a set of evaluation indices, i.e., $X=\{x_{1}, x_{2}, \ldots, x_{N}\}$, where $x_{i}$ is the evaluation index of ATGO in the $i$-th time, while the evaluation index of the competing method is defined as $u$. The *p*-value between ATGO and a competing method with single implementation is calculated by the following two steps:

(1) The statistic value $t_{X}$ is defined as:

$t_{X}=\frac{u_{X}-u}{\sigma_{X}/\sqrt{N}}$ (S3)

where $u_{X}$ and $\sigma_{X}$ are mean and standard deviation for $X$, respectively, and $t_{X}$ obeys t-distribution with degree of freedom = $N-1$ (In this work, $N=10$).

(2) The significance factor (i.e., *p*-value) can be approximately calculated from the probability dense function of t-distribution using integral in ranges of (-$\infty$, -$\left| t_{X} \right|$) and ($\left| t_{X} \right|$, +$\infty$).

We select two-sample t-test [2] to calculate the *p*-value between ATGO and ATGO+, because they are both performed with variable evaluation indices in the entire dataset. Specifically, ATGO and ATGO+ are repeatedly implemented on the benchmark dataset in $N_{1}$ and $N_{2}$ times, respectively, to generate two sets of evaluation indices, i.e., $X=\{x_{1}, x_{2}, \ldots, x_{N_{1}}\}$ and $Y=\{y_{1}, y_{2}, \ldots, y_{N_{2}}\}$, where $x_{i}$ and $y_{j}$ are the evaluation indices of ATGO and ATGO+ in the $i$-th time and $j$-th time, respectively. The *p*-value between ATGO and ATGO+ is calculated by the following two steps:

(1) The statistic value $t_{XY}$ is defined as:

$t_{XY}=\frac{u_{X}-u_{Y}}{\sqrt{\frac{\left( N_{1}-1 \right)S_{X}+\left( N_{2}-1 \right)S_{Y}}{N_{1}+N_{2}-2}(\frac{1}{N_{1}}+\frac{1}{N_{2}})}}$ (S4)

where $u_{X}$ and $u_{Y}$ are mean values for $X$ and $Y$, respectively; $S_{X}$ and $S_{Y}$ are variances for $X$ and $Y$, respectively; and $t_{XY}$ obeys t-distribution with degree of freedom = $N_{1}+N_{2}-2$ (In this work, $N_{1}=N_{2}=10$).

(2) The *p*-value can be approximately calculated from the probability dense function of t-distribution using integral in ranges of (-$\infty$, -$\left| t_{XY} \right|$) and ($\left| t_{XY} \right|$, +$\infty$).

In this work, we use Python package “scipy” to implement Student t-test to calculate the *p*-values in the range from 0 to 1.

**B. Nemenyi post-hoc test.** Given a group of $K$ GO prediction methods, we use $N$ samples (test proteins) to evaluate their performance, where the performance of an individual prediction method is measured by a set of F_1_-scores, each of which is calculated from predicted GO terms and native GO terms on a single sample. For a pair of methods ($M_{i}$, $M_{j}$) in this group, the corresponding *p*-value of F_1_-scores in Nemenyi post-hoc test is calculated by the following three steps.

(1) The difference of average rank between $M_{i}$ and $M_{j}$, denoted as ${DAR}_{ij}$, is calculated:

${DAR}_{ij}=\frac{1}{N}\left| \sum_{n=1}^{N} {rank}_{i,n}-\sum_{n=1}^{N} {rank}_{j,n} \right|$ (S5)

where ${rank}_{i,n}$ is the rank of $M_{i}$ among $K$ methods on the $n$-th sample from the view of F_1_-score.

(2) The statistic value $q_{\alpha}$ is defined as:

$q_{\alpha}={DAR}_{ij}/\sqrt{\frac{K(K+1)}{6N}}$ (S6)

where $q_{\alpha}$ obeys studentized range distribution [3] with degree of freedom = infinity and sample number = $K$. The higher value of $q_{\alpha}$ means the higher significance of performance difference between $M_{i}$ and $M_{j}$, and $\alpha$ is the corresponding significance factor, i.e., *p*-value.

(3) The *p*-value can be approximately calculated from the probability dense function of studentized range distribution with preset statistic thresholds using Gleason’s approach [4].

In this work, we use Python package “scikit posthocs” to implement Nemenyi post-hoc test, where the *p*-values are approximated using Gleason’s approach. Because the minimal and maximal preset statistic thresholds in Gleason’s approach are 0.001 and 0.900, respectively, the *p*-values can be only approximated in range (0.001, 0.900). If the *p*-value is below to 0.001 (or upon 0.900), “scikit posthocs” package will directly output 0.001 (or 1.000).

By reviewing Student t-test and Nemenyi post-hoc test, the big gap between their *p*-values is mainly attributed to the following two aspects. (1) Student t-test can approximate the *p*-values in the range from 0 to 1, while Nemenyi post-hoc test can only approximate the *p*-values in a much smaller range, i.e., (0.001, 0.9), in our programs. (2) The significance of performance difference between two GO prediction methods ($M_{i}$ and $M_{j}$) may be decreased with the increase of the number of methods in a group under Nemenyi post-hoc test. Specifically, we suspect that the significant difference between $M_{i}$ and $M_{j}$ (i.e., the value of $q_{\alpha}$) may be decreased with the increase of the value of $K$, because the increase rate of ${DAR}_{ij}$ is lower than that of $\sqrt{K(K+1)/6N}$ in Eq. S6. To further demonstrate this point, we designed the following test.

Starting from a group of GO prediction methods (named Group A) including SAGP and ATGO, we incrementally add PPIGP, NGP, DeepGO, FunFams, DeepGOCNN, DIAMONDScore, TALE, DeepGOPlus, TALE+, and ATGO+ to Group A and then perform Nemenyi post-hoc test for Group A on our constructed 1068 test proteins in MF aspect. S2 Table lists the statistic values between SAGP and ATGO versus the increase of $K$. It can be found that the *p*-value between SAGP and ATGO is consistently increased from 1.0e-03 to 2.6e-01 (when $2\leq K\leq7$) and then fluctuates in the range from 1.7e-01 to 3.0e-01 (when $8\leq K\leq12$). This observation can be explained as follows. By reviewing Table 1 in the main text, we find that the first five GO prediction methods (i.e., PPIGP, NGP, DeepGO, FunFams, and DeepGOCNN) shows much lower F_max_ values both than SAGP and ATGO in MF aspect, indicating the rank difference between SAGP and ATGO from the view of F_1_-score cannot be changed on most test proteins after adding these five methods into Group A. As a result, the increase rate of DAR is lower than that of $\sqrt{K(K+1)/6N}$, leading to the continuous decrease of $q_{\alpha}$. As for other five methods (DIAMONDScore, TALE, DeepGOPlus, TALE+, and ATGO+), most of them achieve the comparable F_max_ values with both SAGP and ATGO, indicating that the value of DAR will be dramatically increased after adding these methods into Group A. Therefore, the increase of DAR can keep up with that of $\sqrt{K(K+1)/6N}$, leading to the fluctuation of $q_{\alpha}$ in a fixed range.

This experiment has demonstrated two points. Frist, the significance of performance difference between two GO prediction methods in a group is not only dependent on their performance but also associated with the performance of other prediction methods under Nemenyi post-hoc test. Second, this significance may be decreased with the increase of the number of GO prediction methods in a group.

In light of the above data and insight, we prefer to use Student t-test to identify the performance difference between two GO prediction methods at the entire dataset level, because the corresponding *p*-value can be approximated in a more precise range and not be affected by the performances of other prediction methods in the same group.

**Reference**

1. Crawford J, Howell DC, Garthwaite PH. Payne and Jones revisited: estimating the abnormality of test score differences using a modified paired samples t test. Journal of clinical and experimental neuropsychology. 1998; 20:898-905.

2. Heeren T, D'Agostino R. Robustness of the two independent samples t‐test when applied to ordinal scaled data. Statistics in medicine. 1987; 6:79-90.

3. Kokoska S, Nevison C. Critical values for the studentized range distribution. Statistical tables and formulae: Springer; 1989. p. 64-6.

4. Gleason JR. An accurate, non-iterative approximation for studentized range quantiles. Computational statistics & data analysis. 1999; 31:147-58.
