# Supplementary material for: Integrating unsupervised language model with triplet neural networks for protein gene ontology prediction
Source: PLoS Comput Biol. 2022 Dec 22;18(12):e1010793. doi: 10.1371/journal.pcbi.1010793 (PMC9822105; doi:10.1371/journal.pcbi.1010793)
Supplement: S6 Text — (DOCX) [file pcbi.1010793.s026.docx]

**S6 Text. Information content-weighted maximum F_1_-score**

The information content-weighted maximum F_1_-score (ICW-F_max_) is defined as:

$ICW-F_{\max}=\max_{0\leq t\leq1} [\frac{2\cdot icwpr\left( t \right)\cdot icwrc\left( t \right)}{icwpr\left( t \right)+icwrc\left( t \right)}]$ (S7)

where *t* is a cut-off value of confidence score; $iwcpr(t)$ and $icwrc(t)$ are IC-weighted precision and IC-weighted recall, respectively, with confidence score $\geq t$:

$\left\{ \begin{matrix} icwpr\left( t \right)=\frac{\sum_{{GO}_{i}\in GOSET\_TP(t)} IC\left( {GO}_{i} \right)}{\sum_{{GO}_{j}\in(GOSET\_TP(t)\cup GOSET\_FP(t))} IC\left( {GO}_{j} \right)} \\ icwrc\left( t \right)=\frac{\sum_{{GO}_{i}\in GOSET\_TP(t)} IC\left( {GO}_{i} \right)}{\sum_{{GO}_{j}\in(GOSET\_TP(t)\cup GOSET\_FN(t))} IC\left( {GO}_{j} \right)} \end{matrix} \right.$ (S8)

$IC\left( {GO}_{i} \right)=-{log}_{2}(1/p({GO}_{i}|parents of {GO}_{i} in GO))$ (S9)

where $GOSET\_TP(t)$ is the set of correctly predicted GO terms, $GOSET\_TP(t)\cup GOSET\_FP(t)$ is the set of all predicted GO terms, $GOSET\_TP(t)\cup GOSET\_FN\left( t \right)$ is the set of experimentally annotated GO terms, $IC\left( {GO}_{i} \right)$ is the information content for the GO term ${GO}_{i}$, $p({GO}_{i}|parents of {GO}_{i} in GO)$ is the conditional probability of ${GO}_{i}$ given its parents of the GO structure (see details in [1]).

**Reference**

1. Clark WT, Radivojac P. Information-theoretic evaluation of predicted ontological annotations. Bioinformatics. 2013; 29:i53-i61.
