# Supplementary material for: Integrating unsupervised language model with triplet neural networks for protein gene ontology prediction
Source: PLoS Comput Biol. 2022 Dec 22;18(12):e1010793. doi: 10.1371/journal.pcbi.1010793 (PMC9822105; doi:10.1371/journal.pcbi.1010793)
Supplement: S7 Text — (DOCX) [file pcbi.1010793.s027.docx]

**S7 Text. Performance comparison between SAGP and BLAST baseline used in CAFA challenge**

In BLAST baseline of CAFA challenge, the confidence score that a query is associated with GO term $q$ is calculated by:

${S\left( q \right)}_{BLAST-baseline}=max\{s_{1}\cdot{I\left( q \right)}_{1},s_{2}\cdot{I\left( q \right)}_{2},\ldots, s_{n}\cdot{I\left( q \right)}_{n}\}$ (S10)

$s_{i}=N_{i}^{id}/N_{i}^{al}$ (S11)

where *n* is the number of templates in BLAST search, $s_{i}$ is the local sequence identity between query and the *i*-th template, $N_{i}^{id}$ is the number of identical residues in the local alignment region, and $N_{i}^{al}$ is the length of local alignment region; if the *i*-th template is associated with GO term $q$ in the native annotation, ${I\left( q \right)}_{i}=1$; otherwise, ${I\left( q \right)}_{i}=0$.

S11 Table summarizes the performance of SAGP and BLAST baseline on our constructed test dataset and CAFA3 test dataset with different cut-off values of sequence identity. It can be found that SAGP achieves much better performance than BLAST baseline in all three GO aspects. Taking CAFA3 test dataset as an example, SAGP gains 54.9% and 65.8% average improvements of F_max_ and AUPR values, respectively, in three GO aspects under the cut-off $t_{1}=30\%$. In addition, BLAST baseline shows much worse performance than most of competing methods, such as FunFams and DeepGOPlus.
