# Supplementary material for: Integrating unsupervised language model with triplet neural networks for protein gene ontology prediction
Source: PLoS Comput Biol. 2022 Dec 22;18(12):e1010793. doi: 10.1371/journal.pcbi.1010793 (PMC9822105; doi:10.1371/journal.pcbi.1010793)
Supplement: S8 Text — (DOCX) [file pcbi.1010793.s028.docx]

**S8 Text. Performance comparison between four metric learning methods**

We separately use four metric learning methods, including F_1_-score (F_1_, see Eq. S32 in S10 Text), Jaccard similarity (JS) [1], weighted F_1_-score (WF_1_), and weighted Jaccard similarity (WJS), to measure the functional similarity in triplet loss, where the weights of GO terms are measured by information content [2]. The formulas of JS, WF_1_, and WJS are described as follows.

$JS=\frac{\left| {GOSET}_{A}\cap{GOSET}_{B} \right|}{\left| {GOSET}_{A}\cup{GOSET}_{B} \right|}$ (S12)

$WF_{1}=2({p\mathrm{re}}_{w}\times\mathrm{rec}_{w})/({p\mathrm{re}}_{w}+\mathrm{rec}_{w})$ (S13)

${p\mathrm{re}}_{w}=\frac{\sum_{{GO}_{i}\in{(GOSET}_{A}\cap{GOSET}_{B})} w({GO}_{i})}{\sum_{{GO}_{j}\in{GOSET}_{A}} w({GO}_{j})}$, $\mathrm{rec}_{w}=\frac{\sum_{{GO}_{i}\in{(GOSET}_{A}\cap{GOSET}_{B})} w({GO}_{i})}{\sum_{{GO}_{j}\in{GOSET}_{B}} w({GO}_{j})}$ (S14)

$\mathrm{WJS}=\frac{\sum_{{GO}_{i}\in{(GOSET}_{A}\cap{GOSET}_{B})} w({GO}_{i})}{\sum_{{GO}_{j}\in{(GOSET}_{A}\cup{GOSET}_{B})} w({GO}_{j})}$ (S15)

$w\left( {GO}_{i} \right)=-{log}_{2}(1/p({GO}_{i}|parents of {GO}_{i} in GO))$ (S16)

where ${GOSET}_{A}$ and ${GOSET}_{B}$ are sets of GO terms in native annotations for proteins A and B, respectively, $\left| . \right|$ is the number of elements in a set, $w({GO}_{i})$ is the weight (measured by information content) of ${GO}_{i}$, and $p({GO}_{i}|parents of {GO}_{i} in GO)$ is the conditional probability of ${GO}_{i}$ given its parents of the GO structure (see details in [3]). Two proteins are considered to have the same function if their functional similarity is larger than a cut-off value $c_{f}$. The values of $c_{f}$ are 0.8, 0.5, 0.8 and 0.5 for F_1_, JS, WF_1_, and WJS, respectively, in each GO aspect.

For each metric learning method, we re-trained the corresponding GO prediction model using the ATGO framework, which was further benchmarked on our constructed test dataset and CAFA3 test dataset, as summarized in S12 Table. We found that there is no significant performance difference among four metric learning methods for each GO aspect. Specifically, the absolute increases of F_max_ values between the best and worst performers are both less than 0.01 for all three aspects in each test dataset, suggesting that the effectiveness of the proposed ATGO framework is not sensitive to the choices of different metric learning methods.

**Reference**

1. Bag S, Kumar SK, Tiwari MK. An efficient recommendation generation using relevant Jaccard similarity. Information Sciences. 2019; 483:53-64.

2. Hayn C. The information content of losses. Journal of accounting and economics. 1995; 20:125-53.

3. Clark WT, Radivojac P. Information-theoretic evaluation of predicted ontological annotations. Bioinformatics. 2013; 29:i53-i61.
