# Supplementary material for: Integrating unsupervised language model with triplet neural networks for protein gene ontology prediction
Source: PLoS Comput Biol. 2022 Dec 22;18(12):e1010793. doi: 10.1371/journal.pcbi.1010793 (PMC9822105; doi:10.1371/journal.pcbi.1010793)
Supplement: S9 Text — (DOCX) [file pcbi.1010793.s029.docx]

**S9 Text. The mathematics formulas for ESM-1b transformer**

**A. Masking**

For an input sequence, the masking strategy [1] is performed on the corresponding tokens (i.e., amino acids). Specifically, we randomly sample 15% tokens, each of which is changed as a special “masking” token with 80% probability, a randomly-chosen alternate amino acid with 10% probability, and the original input token (i.e., no change) with 10% probability.

**B. One-hot encoding**

The masked sequence is represented as a $L\times28$ matrix using one-hot encoding [2], where 28 is the types of tokens, including 20 common amino acids, 6 non-common amino acids (B, J, O, U, X and Z), 1 gap token, and 1 “masking” token.

**C. Embedding with positions**

The one-hot coding matrix $X$ of the masked sequence is multiplied by an embedding weight matrix $W_{E}$ to generate an embedding matrix $H_{E}$:

$H_{E}=XW_{E}, X\in R^{L\times28}, W_{E}$ $\in R^{28\times D}, H_{E}$ $\in R^{L\times D}$ (S17)

where $L$ is the length of the masked sequence, 28 is the types of tokens in the masked sequence, and $D$ is the embedding dimension.

Then, the position embedding strategy is used to record to position of each token in the masked sequence to generate a position embedding matrix $H_{P}$:

$H_{P}=\left[ \begin{aligned} h_{1} \\ h_{2} \\ \ldots\\ h_{L} \end{aligned} \right], h_{i}=\left( v_{i,1},v_{i,2},{\ldots,v}_{i,D} \right),H_{P}\in R^{L\times D}\text{ , and }h_{i}\in R^{D}$ (S18)

$v_{i,2k}=\sin(\frac{i}{{10000}^{2k/D}}){\text{,} v}_{i,2k+1}=\cos(\frac{i}{{10000}^{(2k+1)/D}})$, $k=0, 1, .., (D-1)/2$ (S19)

where$\text{ }h_{i}$ is the embedding vector for the $i$-th position in the masked sequence.

Finally, two embedding matrices are added as a combination embedding matrix $H_{1}:$

$H_{1}=H_{E}+$ $H_{P}, H_{1}$ $\in R^{L\times D}$ (S20)

**D. Self-attention**

The embedding matrix $H_{1}$is fed to self-attention block with $n$ layers, each of which consists of $m$ attention heads, a linear unit, and a feed-forward network (FFN). In each attention head, the scale dot-product attention is performed as follows:

$A_{i,j}=softmax(M_{i,j}^{Q}{M_{i,j}^{K}}^{T}/\sqrt{d_{ij}}) M_{i,j}^{V}$ (S21)

$M_{i,j}^{Q}=H_{i}W_{i,j}^{Q}$, $M_{i,j}^{K}=H_{i}W_{i,j}^{K},$ $M_{i,j}^{V}=H_{i}W_{i,j}^{V}$ (S22)

$d_{ij}=D/m$, $W_{i,j}^{Q},W_{i,j}^{K},W_{i,j}^{V}$ $\in R^{D\times(\frac{D}{m})}$, $M_{i,j}^{Q}$, $M_{i,j}^{K}$,$M_{i,j}^{V}\text{,} A_{i,j}\in R^{L\times(\frac{D}{m})}$ (S23)

where $A_{i,j}$ is the attention matrix in the ($i$-th layer, $j$-th head), $M_{i,j}^{Q}$, $M_{i,j}^{K}$, and$M_{i,j}^{V}$are Query, Key, and Value matrices in the ($i$-th layer, $j$-th head), $H_{i}$is the input matrix in the $i$-th layer,$W_{i,j}^{Q}$, $W_{i,j}^{K}$, and $W_{i,j}^{V}$are weight matrices, and $d_{ij}$ is the scale parameter.

The outputs of all attention heads in $i$-th layer are concatenated as a new matrix $A_{i}$, which is further fed to a linear unit to output the matrix $U_{i}$:

$A_{i}=A_{i,1}A_{i,2}\ldots A_{i,m}$ (S24)

$U_{i}={A_{i}W}_{i}^{1}+b_{i}^{1}, W_{i}^{1}$ $\in R^{D\times D}, A_{i}, b_{i}^{1},U_{i}\in R^{L\times D}$ (S25)

where $W_{i}^{1}$ and $b_{i}^{1}$ are the weight matrix and bias, respectively, in the linear unit.

**E. Feed-forward network with shortcut connections**

The $U_{i}$ is added by $H_{i}$to generate a new matrix $F_{i}$, which is further fed to the FFN to output the matrix $T_{i}$:

$F_{i}$= $H_{i}$+ $U_{i}$ (S26)

$T_{i}=gelu\left( F_{i}W_{i}^{2}+b_{i}^{2} \right)W_{i}^{3}+b_{i}^{3}$, $W_{i}^{2},W_{i}^{3}$ $\in R^{D\times D}$, $b_{i}^{2},b_{i}^{3},T_{i}\in R^{L\times D}$ (S27)

$\mathrm{gelu}\left( x \right)=x\emptyset(x)$ (S28)

where $W_{i}^{2}$ and $W_{i}^{3}$ are weight matrices in the FFN, $b_{i}^{2}$ and $b_{i}^{3}$ are bias in the FFN, and $\emptyset\left( x \right)$is the integral of Gaussian Distribution for $x$

The $F_{i}$ is added by $T_{i}$ as the output the $i$-th attention layer:

$H_{i+1}$= $F_{i}$+ $T_{i}, H_{i+1}\in R^{L\times D}$ (S29)

The output of the last attention layer is fed to a fully connected layer with SoftMax function to generate a $L\times28$ probability matrix:

$P=SoftMax\left( H^{n}W^{n}+b^{n} \right), P\in R^{L\times28}$ (S30)

where the ($l$-th, $c$-th) value in $P$ indicates the probability that the $l$-th token in the masked sequence is predicted as the $c$-th type of amino acid, $W^{n}$ and $b^{n}$ are weight matrix and bias, respectively.

**F. Loss function**

The loss function is designed as:

${Loss}_{esm}=E_{x\sim X}\sum_{l\in x(M)} \left( -\frac{logP_{l, c\left( l \right)}}{\left| x\left( M \right) \right|} \right)$ (S31)

where $x$ is a sequence in training protein set $X$, $x(M)$ is a set of masking position in $x$, $\left| x(M) \right|$ is the number of elements in $x(M)$, $c(l)$ is the type index of amino acid for the $l$-th token in $x$ before masking, and -$logP_{l, c\left( l \right)}$ is negative log likelihood of the true amino acid $x_{l}$ under condition of masking.

**Reference**

1. Devlin J, Chang M-W, Lee K, Toutanova K. Bert: Pre-training of deep bidirectional transformers for language understanding. arXiv preprint arXiv:181004805. 2018; 1:1-15.

2. Buckman J, Roy A, Raffel C, Goodfellow I, editors. Thermometer encoding: One hot way to resist adversarial examples. International Conference on Learning Representations; 2018; 1-12.
