# Supplementary material for: Integrating unsupervised language model with triplet neural networks for protein gene ontology prediction
Source: PLoS Comput Biol. 2022 Dec 22;18(12):e1010793. doi: 10.1371/journal.pcbi.1010793 (PMC9822105; doi:10.1371/journal.pcbi.1010793)
Supplement: S10 Text — (DOCX) [file pcbi.1010793.s030.docx]

**S10 Text. The functional similarity between two proteins**

The functional similarity of two proteins is measured by the F_1_-score between their GO terms:

$F_{1}-score=2(pre\times rec)/(pre+rec)$, $pre=ns/n_{a}$, $rec=ns/n_{b}$ (S32)

where $ns$ is the number of same GO terms between two proteins, $n_{a}$ and $n_{b}$ are the numbers of GO terms for proteins $a$ and $b$, respectively.
