# Supplementary material for: Integrating unsupervised language model with triplet neural networks for protein gene ontology prediction
Source: PLoS Comput Biol. 2022 Dec 22;18(12):e1010793. doi: 10.1371/journal.pcbi.1010793 (PMC9822105; doi:10.1371/journal.pcbi.1010793)
Supplement: Attachment — Submitted filename: response_to_reviewers.pdf [file pcbi.1010793.s031.pdf]

Response to Reviewer #1

We very much appreciate the comments and suggestions from the Reviewer, which we found very helpful for improving the quality of the manuscript. One of the major concerns was on the lack of interpretation in the observation that the ablative model M1 obtained the highest performance improvement, particularly in MF aspect, in the section of "Ablation study". Accordingly, we have added an explanation to the observation which we believe is associated to the correlation of embedding model and function feature. The Reviewer also raised the concern on the lack of citations on other sequence embedding systems, where we have added reviews on several embedding systems, including ProtTrans, TAPE, and Bepler & Berger's approach, in Introduction. In the following, we include point-by-point replies to the comments of the Reviewer, where all changes have been highlighted in yellow in the manuscript.

1. **<u>The Reviewer commented:</u>**

> ***This work presents a new protein function prediction approach and software. The main idea is to exploit pre-trained language models on protein sequences and use them as a basis for downstream tasks of GO term prediction. The paper is clear and well written. The architecture makes sense, the loss function for training is interesting, and the results were thorough and favor their method. Ultimately, the authors find a need to specifically combine homology-based models with network-based models.***

We appreciate the positive comments from the Reviewer on the work.

2. **<u>The Reviewer commented:</u>**

> ***I have some questions and concerns, mostly minor:***
>
> **** The paper works off of the ESM model, however, there are other embedding systems including ProtTrans (https://arxiv.org/abs/2007.06225), TAPE (https://arxiv.org/abs/1906.08230), and Bepler & Berger's approach (https://arxiv.org/abs/1902.08661). Some mention or comments on the value of those systems would be important to make.***

This is an excellent point. Following Reviewer's suggestions, we have added a new paragraph in Introduction to review several other embedding systems developed in the field, including ProtTrans, TAPE, Bepler & Berger's approach, and ESM-1b transformer (Page 4):

> To alleviate the issue caused by the lack of annotated data, a promising approach is to utilize protein language models pre-trained through deep-learning networks on large-scale sequence databases which may not have functional annotations. Due to the extensive sequence training and learning, important inter-residue correlation patterns, which are critical for protein functions, can be extracted through the language models and utilized for functional embedding. Several protein language models, such as ProtTrans [23], models used in TAPE [24], and Bepler & Berger's approach [25], have been recently proposed in the literature. Especially, an unsupervised protein language model, ESM-1b transformer [26], which utilized self-attention networks to learn the evolutionary diversity from 250 millions of protein sequences, has found impressive usefulness in protein contact-map and structure prediction.

3. **<u>The Reviewer commented:</u>**

> ***The ProtTrans paper find their models to be superior to ESM-1b. If so, this could propagate to this paper and further improvements might be possible.***
>
> ****** The ablative models showed interesting performance in that M1 obtained the highest improvement in accuracy and the addition of other components only had marginal effect, particularly in MF though the trend is the same in BO and CC (Figure 5). Would other embedding methods show similar performance gain or there is something special with ESM-1b?***

We appreciate the Reviewer's question on whether the use of a different sequence embedding method such as ProtTrans could potentially improve GO prediction. Given that these language models used similar modeling approaches, we assumed that they should have grasp similar embedding features and their impacts on the function annotation predictions are probably similar. While we agree with the Editor that a detailed comparative investigation on different language models can be expensive and out of scope of this work, we will leave it for future study. Accordingly, we added the following paragraph to discuss the potential (Page 12):

> Despite the demonstrated effectiveness of the transformer, it is important to note that the currently used ESM-1b transformer is only one of the several existing language models built on a single query sequence. The use of other embedding language models such as ProtTrans and a newly released extended version ESM-2 [42], both of which demonstrated superiority to ESM-1b, for GO prediction is worthy of exploration in future work.

4. **<u>The Reviewer commented:</u>**

> ****** Is there some interpretation in that most gain comes from the embeddings for MF. This suggest that embeddings contain some sequence signal. Have the authors tried to understand this aspect?***

Thank you for raising the question. Following the suggestions from the Reviewer, we have added the following paragraph to explain the above-mentioned observation (Page 10):

> Compared to other function categories, M1 achieves the highest performance improvement in MF prediction, indicating that the embeddings from ESM-1b contains sequence signals which have a closer relationship with MF than with BP and CC. This is probably because MF is mainly associated with the molecular action that a protein can perform by itself and therefore directly related to the internal feature of the sequence that can be captured by the sequence embedding. On the other hand, BP describes the biological pathways which often involve a series of intermolecular actions. Rather than a single protein, a group of interacting proteins are required to complete a BP. Since the sequence embedding is primarily derived from the sequence itself and not designed for capturing the information of protein-protein interactions or co-expression regulations, it is less effective for BP prediction. Similarly, the CC of a protein such as subcellular localization is dependent on other molecules in the cell and cannot be fully captured by the embedding.

5. The Reviewer commented:

> ***** The paper is written by mixing past and present tense. The authors can decide to use one. For example, "...AGTO+ achieved a further improvement overall three categories of GO terms. It also significantly outperforms..." is an example of mixing tenses. Also, "overall" -> "over all"***

Thank you for pointing out the issue. We have read through the manuscript and double-checked and corrected the tenses. The above-mentioned sentence has been rewritten as following (Page 5):

> After combining ATGO with SAGP, the composite AGTO+ achieves a small but consistent improvement over all three categories of GO aspects in terms of Fmax and AUPR, where the *p*-values in Student's test between them are both below 4.3e-02, showing the difference is statistically significant at the entire dataset level. ATGO+ also significantly outperforms the two composite control methods, DeepGOPlus and TALE+, with *p*-values below 8.4e-09 in all the comparisons, although these two control methods clearly outperform their corresponding single-based methods (DeepGOCNN and TALE) respectively.

6. The Reviewer commented:

> ***** life mechanism -> life mechanisms (2 places)***

Thank you for pointing out the issue. We have corrected "life mechanism" as "life mechanisms" in Pages 1 and 3 of main text, as following.

> Accurate identification of protein function is critical to elucidate life mechanisms and design new drugs.

> To elucidate life mechanisms, it is critical to identify the biological functions of proteins, which have been grouped, in the context of the widely used Gene Ontology (GO), into three aspects of molecular function (MF), biological process (BP), and cellular component (CC) [2].

Response to Reviewer #2

We very much appreciate the comments and suggestions from the Reviewer, which we found very helpful for improving the quality of the manuscript. The Reviewer raised the concern on the lack of interpretation in the use of three embedding components in the proposed ATGO architecture. To address this issue, we have added the rationale and experimental evidence that supports the choice of the utilization of three embedding layers from language models. In the following, we included point-by-point replies to the comments of the Reviewer, where all changes have been highlighted in yellow in the manuscript.

1. **<u>The Reviewer commented:</u>**

> ***This manuscript reports a new deep learning method to predict the function (GO terms) of proteins. The MSA embedding techniques based on self-attend are first applied to this problem. The method performs better than both control methods and other state-of-the-art methods on the CAFA3 benchmark. Therefore, the work makes valuable contributions to the field.***

We appreciate the positive comments from the Reviewer on the work.

2. **<u>The Reviewer commented:</u>**

> ***There is some minor issue regarding the deep learning architecture. It is not clear why three embedding components (triplet networks) are used. Some rationale should be provided to justify the design of the architecture.***

Thank you for the helpful suggestion. The purpose of using three embedding components in the proposed ATGO architecture is to relieve the negative impact caused by information loss in feature extraction. Specifically, different layers of a protein language model such as ESM-1b capture different views of information and the layer closer to the end of the network can extract more abstract information. Considering that the feature embedding from a single view (layer) cannot fully represent the evolutionary information for sequence, we extract feature embeddings from multiple views (i.e., the last three layers of ESM-1b) to relieve information loss, as described in the Page 13:

> Considering that the feature embedding from a single view (layer) cannot fully represent the evolutionary information for sequence, we extract feature embeddings from multiple views (i.e., the last three layers) to relieve information loss.

Moreover, we have examined the result of using three embedding components in the experiment of "Ablation study", which further demonstrated the advantage of choice. Specifically, although the ablative models M2 and M3 use the same ESM-1b model for feature extraction and the same loss function for training, M3 still outperforms M2 by using last three rather than just the last one layer of ESM-1b for feature extraction (see Figures 5 and S5). This is partly because M3 captures more fine-grained information from the different layers of ESM-1b than M2. Accordingly, we have added the following paragraph to summarize the insight (Page 10):

Although M2 and M3 use the same ESM-1b model for feature extraction and the same loss function for training, M3 still slightly outperforms M2 by using the last three rather than just the last one layer of ESM-1b for feature extraction. This is partly because that different layers of a transformer model such as ESM-1b capture different levels of information and the layers closer to the end of the transformer tend to extract more abstract information. Therefore, M3 captures more fine-grained information from ESM-1b than M2.

Response to Reviewer #3

We very much appreciate the comments and suggestions from the Reviewer, which we found very helpful for improving the quality of the manuscript. One of the major concerns is the suitability of F1-score as a learning metric in the proposed ATGO framework. Accordingly, we have added a new paragraph to benchmark four metric learning methods, including F1-score and other three methods suggested by Reviewer, on two test datasets, where we found that there is no significant performance difference among four methods. The Reviewer also raised a concern on the lack of proper statistical test for performance comparison between some method pairs. To address this issue, we performed two types of statistical tests, including Student's t-test *at the entire dataset level* and Friedman-Nemenyi test *at the individual protein level*, where the latter test allows us to examine the difference among all GO prediction methods. In addition, we have added interpretations and further clarifications on two seemingly confusing experimental observations, including the statistical significance between ATGO and ATGO+ and the difference between the SAGP and BLAST baseline used in CAFA. In the following, we include point-by-point replies to the comments of the Reviewer, where all changes have been highlighted in yellow in the manuscript.

1. <u>The Reviewer commented:</u>

> ***The authors propose deep learning model (ATGO) for automatically assigning GO terms to protein sequences. The model uses a pre-trained language model to extract three different protein embedding vectors which are further combined into one vector using a neural network. A triplet loss is used to enforce that proteins with similar functions are close in the embedding space. The model and a combination of the model with homology searching are compared to several other well-known function prediction methods on two datasets.***

We appreciate the positive comments from the Reviewer on the work.

2. <u>The Reviewer commented:</u>

> ***Major comments***
> ***1) A main conclusion of the study is that the use of a pre-trained protein language model is by far the main source of performance for this model. However, the superiority of these language models with respect to supervised learners such as CNNs has already been extensively demonstrated (also for the CAFA3 dataset) in the past. See Rao et al. Adv Neural Inf Process Syst. 2019, Littmann et al. Scientific Reports, 2021, Villegas-Morcillo et al., Bioinformatics (2021).***

We thank the Reviewer for bringing to our notice these other studies of protein language models. We have added a new paragraph in Introduction to review these efforts (see Page 4):

> To alleviate the issue caused by the lack of annotated data, a promising approach is to utilize protein language models pre-trained through deep-learning networks on large-scale sequence databases which may not have functional annotations. Due to the extensive sequence training and learning, important inter-residue correlation patterns,

which are critical for protein functions, can be extracted through the language models and utilized for functional embedding. Several protein language models, such as ProtTrans [23], models used in TAPE [24], and Bepler & Berger's approach [25], have been recently proposed in the literature. Especially, an unsupervised protein language model, ESM-1b transformer [26], which utilized self-attention networks to learn the evolutionary diversity from 250 millions of protein sequences, has found impressive usefulness in protein contact-map and structure prediction. Meanwhile, the unsupervised language models have also been used, often through supervised learners such as convolutional neural networks (CNNs), for protein function prediction tasks, with examples including the predictions of protein molecular function [27], mutation and stability [24], subcellular localization [23], GO transferals [28], and ligand binding [29].

3. The Reviewer commented:

2) The idea of using metric learning to learn an embedding space that reflects structural similarity is interesting (although it has also been done before for molecular function prediction for enzymes – Aman Memon et al., Bioinformatics, 2020). However, the authors here calculate the F1 score between the annotations of two proteins and pairs with F1 larger than a threshold are considered positive. But the F1 score is not a proper distance metric (e.g. doesn't satisfy triangle inequality) and this might hamper the performance of the triplet loss. See Powers Technical report KIT-14-001 for more information on the F1 score. Perhaps a Jaccard similarity (even weighted by term information content?) might be a more suitable metric to use for metric learning.

Thank you for raising the interesting point. Following the suggestions, we re-trained our models using four different metrics, including F1-score, Jaccard similarity, weighted F1-score, and weight Jaccard similarity, to assess the function similarity in the proposed ATGO framework, where the weights of GO terms are measured by information content. The resultant ATGO models via the four metric learning methods are benchmarked on our constructed dataset and CAFA3 dataset, with results summarized in Table S12. It was found, however, that there is no obvious difference on the overall performance among the four metric learning methods in each GO aspect, suggesting that the effectiveness of the proposed ATGO framework is not sensitive to the selection of specific metrics. Therefore, we keep using the F1-score as the metric in the ATGO framework. We added the following paragraph to summarize the insight (Page 11):

In addition, to examine the impact of different metric learning methods on the ATGO model (see "Methods and Materials"), we used four metrics of F1-score, Jaccard similarity [40], weighted F1-score, and weighted Jaccard similarity to assess the functional similarity in the triplet loss separately, where the weights of GO terms are measured by information content [41]. The performance of the ATGO models via the four metric learning methods on our test datasets is summarized in Table S12 and discussed in Text S8. It is found that although the performance of individual models varies, there is no obvious difference on the overall performance of the models for each GO aspect, suggesting that the effectiveness of the proposed ATGO framework is not sensitive to the choices of different metric learning methods.

We also added a citation to the work by SA Memon et al on the triplet network (see Ref [62]).

4. The Reviewer commented:

> ***3) The conclusion that combining ATGO with sequence homology boosts performance is not sufficiently supported. First, by looking at Tables 1 and 2, the performance improvement is in most cases <0.01 and the coverage of ATGO is already 100%. Figure 6 does show that for one protein there is a BLAST hit that helps detect some functions that were missed by the network, but looking at the numbers, this seems to be a rare occasion. So given the very small gain and that for some proteins ATGO+ can do worse than ATGO (top of Fig 6), the conclusion that information fusion leads to better performance for ATGO is not valid I think.***

We agree that the overall improvement brought from sequence homology is not dramatic for ATGO. However, the improvement is consistently seen in all our test sets, as well as other methods (TALE and DeepGOCNN), showing that the sequence homology-based transferal is indeed complementary to the deep-learning models and the overall improvement is robust. Accordingly, we have weakened the statement and modified the conclusion from "…the composite AGTO+ achieves a further improvement…" to "…the composite AGTO+ achieves a slight improvement…". We also added an explanation on the difference in magnitude of improvement brought by the sequence homology for different base methods (Page 5):

> After combining ATGO with SAGP, the composite AGTO+ achieves a small but consistent improvement over all three categories of GO aspects in terms of Fmax and AUPR, where the *p*-values in Student's test between them are both below 4.3e-02, showing the difference is statistically significant at the entire dataset level. ATGO+ also significantly outperforms the two composite control methods, DeepGOPlus and TALE+, with *p*-values below 8.4e-09 in all the comparisons, although these two control methods clearly outperform their corresponding single-based methods (DeepGOCNN and TALE) respectively. It cannot escape our notice that the magnitude of performance difference between ATGO and ATGO+ is smaller than that between TALE and TALE+ (or between DeepGOCNN and DeepGOPlus) in each GO aspect as shown in Table 1. Part of the reason is that ATGO by itself is a much more accurate predictor compared to other deep learning predictors such as DeepGO and TALE. Therefore, adding another component of sequence homology can bring a relatively smaller increase in the overall Fmax and AUPR scores. In fact, ATGO alone already outperforms both DeepGOPlus and TALE+, which provides a solid base for the better performance of AGTO+. Nevertheless, a consistent improvement has been seen in all datasets and approaches when adding the homology transferal component, demonstrating the advantage of combining different sources of information for improving protein function predictions.

As pointed out by the Reviewer, the coverage of ATGO already reaches 100%. Therefore, we modified the conclusion "…could further improve the coverage and accuracy of…" to "…could further improve the accuracy of …", as described in Pages 1, 4, and 12.

> In addition, it was found that the combination of the network scores with the complementary homology-based inferences could further improve the accuracy of the predicted models.
>
> To improve prediction accuracy, we further implemented a composite version, ATGO+, by combining ATGO with a sequence homology-based model.
>
> Finally, combining ATGO with complementary information from sequence

homologous inference can further improve the prediction accuracy.

5. The Reviewer commented:

4) It is a bit strange that the second best method in the CAFA experiment is the blast-like baseline, although several methods that participated in CAFA3 and that were developed later (e.g. DeepGOPLus) perform considerably better than the BLAST baseline in this dataset.

Thank you for raising the point. The SAGP (sequence alignment-based GO prediction) method used in this study is a newly developed method built on BLAST alignments which is different from the BLAST baseline method used in CAFA challenge. The main difference between SAGP and BLAST baseline of CAFA is that the former deduces the functional patterns from multiple homology templates (see Eq. S1 in SI) while the latter only uses a single template for function deduction (see Eq. S10). Therefore, SAGP inherits more functional knowledge from homology templates based on consensus, compared to the BLAST baseline. We further compared SAGP with BLAST baseline in our constructed test dataset (1068 non-redundant proteins) and CAFA3 test dataset, with results summarized in Table S11 and discussed in Text S7. It was shown that BLAST baseline shows much worse performance than SAGP and other competing methods, such as FunFams and DeepGOPlus.

To clarify this point, we have added a new paragraph of main text to discuss the comparison of BLAST baseline of CAFA with SAGP and other competing methods (Page 9):

> Interestingly, SAGP outperforms most of other control methods. Although both are built on BLAST alignments, SAGP is different from the BLAST baseline method used in CAFA challenge [33]; the main difference between these two is that the former deduces consensus functional patterns from multiple homology templates (see Eq. S1 in SI) while the latter only uses a single template for function deduction (see Eq. S10). In Table S11, we further compare SAGP with the CAFA BLAST baseline in our constructed test dataset (1068 non-redundant proteins) and CAFA3 test dataset, respectively, with details explained in Text S7. It is shown that SAGP significantly outperforms the CAFA BLAST baseline, suggesting the importance of combining multiple templates in the homology-based protein function inference. Consistently with the CAFA experiments [33], the BLAST baseline also underperforms other competing methods, such as FunFams and DeepGOPlus, as shown in Tables 1, 2 and S11.

6. The Reviewer commented:

5) The p-value calculations are a little problematic. First, it is not clear how the authors controlled for multiple comparisons. Additionally they don't compare all against all, but rather ATGO against all and ATGO+ against all, but not ATGO against ATGO+ (this is related to comment 2 as well). Other comparisons such as between SAGP and PPIGP are done implicitly in the manuscript without the proper statistical test. Ideally the authors should use analysis of variants to identify whether one or multiple methods is significantly better than the rest and then use appropriate post-hoc tests to perform the pairwise comparisons.

Thank you for this valuable suggestion. In Tables 1 and 2, we identified the performance difference between GO prediction methods *at the entire dataset level*, where the prediction performance was measured by Fmax and AUPR values. Specifically, all of 12 GO prediction methods were divided into two groups, including 9 single methods and 3 composited methods, where ATGO and ATGO+ were compared with other 8 single methods and 2 composited methods, respectively. In each of the pairwise comparisons, the proposed method (ATGO or ATGO+) was repeatedly implemented on the benchmark datasets for 10 times to generate the corresponding performance evaluation indices (Fmax and AUPR values), which were compared with the fixed evaluation index generated by the competing method to calculate *p*-value using two-sided Student's t-test. One reason for us to divide the comparisons into two groups is that we want to highlight the difference between single methods and the difference between composite methods, separately, as a comparison between composite and single methods can be less specific and insightful. To clarify this point, we have added an explanation of the pairwise comparison in the captions of Tables 1, 2, S3, and S4, as follows.

> Specifically, the proposed ATGO and ATGO+ are repeatedly implemented with 10 times on the benchmark dataset to generate the corresponding performance evaluation indices, which are compared with the fixed evaluation index generated by the competing method to calculate *p*-value using two-sided Student's t-test.

Since most of the competing methods, such as SAGP and PPIGP, were performed with constant Fmax and AUPR values in the entire dataset, there was no proper statistical test presented for the corresponding pairwise comparison. However, considering this barrier at the entire dataset level comparison and following the suggestion from the Reviewer, we performed a new set of analyses of variants and post-hoc test to identify the performance difference of GO prediction methods *at the individual protein level*, where the performance of each prediction method is measured by a set of F1-scores, each of which is calculated from the predicted GO terms and native GO annotation in a single protein. Specifically, we firstly use Friedman test, one of the most used approaches in analysis of variants, to examine whether there is a significant performance difference among 12 GO prediction methods. If the significance factor (i.e., *p*-value) is below to 0.05 in Friedman test, the Nemenyi post-hoc test is further performed to identify the performance difference between pairwise comparisons.

Accordingly, we have added the following paragraph to discuss the all-to-all pairwise comparison results between 12 methods on the 1068 benchmark proteins, with the statistics method and the data results presented in Text S4 and Table S1 in SI respectively (Page 5):

> It is noted that the Student's t-test *p*-values in Table 1 are calculated by comparing 10 independent implementations of ATGO/ATGO+ to a single implementation of the competing methods. To examine the statistical significance between all individual methods, most of which have only single implementation, we perform Friedman test [35], one of the most used approaches in analysis of variants, for the 12 GO prediction methods at individual protein level (see details in Text S4). A significant performance

difference with p-values ≤4.6e-106 is found among all prediction methods in three GO aspects. Thus, a Nemenyi post-hoc test [36] is performed to calculate the p-value of performance difference between each pair of prediction methods, with result listed in Table S1. It can be found that the p-value between the propose ATGO/ATGO+ and each of competing methods is below 0.05 in each GO aspect *at the individual protein level*, except for (ATGO, SAGP), (ATGO, DeepGOPlus), and (AGTO+, SAGP) in MF with the p-values of 2.5e-01, 3.0e-01, and 1.5e-01, respectively. However, the p-values of Fmax values for the above-mentioned three pairwise methods in MF are 1.1e-07, 1.5e-06, and 4.8e-11, respectively, under Student's t-test *at the entire dataset level*, while the corresponding p-values for AUPR values are 3.1e-17, 1.1e-11, and 1.6e-18, respectively, as shown in Table 1. Meanwhile, although the difference between ATGO and ATGO+ is not significant at the individual protein level with the Nemenyi post-hoc test p-value>=0.90 for three GO aspects, the difference is statistically significant at the entire dataset level, with the Student's t-test p-value=1.3e-03/1.2e-12/3.2e-05 for Fmax and 1.8e-09/3.9e-12/4.3e-02 for AUPR on MF/BP/CC terms respectively. Part of the reason for the p-value difference in the two calculations is that *at the individual protein level* ATGO/ATGO+ are measured by a set of F1-scores on hundreds of proteins with significant functional differences while *at the entire dataset level* the ATGO/ATGO+ are run at a fixed index obtained through the average of confidences scores of 10 models, where each ATGO+ model consistently show higher evaluation index than the corresponding ATGO model. Another reason is that the Student's t-test is mathematically different from that in the Nemenyi post-hoc test (see explanation in Table S2 and Text S5). Given that the Student's t-test can be approximated in a more precise range and not be affected by the performances of other prediction methods in the same group (see details in Text S5), we present the results mainly on the t-test in this work.

Similarly, for the CAFA3 test dataset, the p-values between the propose ATGO/ATGO+ and competing methods are both below 0.05 in all three GO aspects by Nemenyi post-hoc test, as summarized in Table S6. Accordingly, we have added the following paragraph to discuss the Friedman and Nemenyi post-hoc tests in the CAFA3 test dataset, as described in Pages 8.

To examine the pairwise significance, we perform Friedman test [35] for the 10 GO prediction methods at the individual protein level and found that there exists a significant performance difference with p-value ≤6.0e-168 in each GO aspect. Next, the Nemenyi post-hoc test [36] is further performed to calculate the p-values of performance difference between all method pairs. As shown in Table S6, the p-values between ATGO/ATGO+ and all the competing methods are both below 0.05 in three aspects at the individual protein level.

7. <u>The Reviewer commented:</u>

Minor comments
1) It would be nice to include an information theory-based evaluation metric such as semantic distance or IC-weighted Fmax. The number of positive examples does not always mean that the term is difficult to predict, depending on how many 'sibling' terms exist

Thank you for this valuable suggestion. Following the suggestion, we have added an information theory-based evaluation metric, i.e., information content-weighted maximum F1-score (ICW-Fmax, see Text S6 of SI), to measure the prediction performance of the proposed ATGO/ATGO+ and other competing methods on our constructed test dataset and CAFA3 test dataset, which are summarized in Tables S5

and S7. Experimental results showed that both ATGO and ATGO+ outperform each of competing methods based on the new metric score.

Accordingly, we added two new paragraphs to discuss the performance comparison between ATGO/ATGO+ and other competing methods with respect to ICW-Fmax values (Pages 7 and 8 respectively):

> We further assess the modeling results using an information theory-based evaluation metric, i.e., information content-weighted maximum F1-score (ICW-Fmax), which is defined by Eq. S7 in Text S6. The results for 12 GO prediction methods on the 1068 test proteins are listed in Table S5 of SI, which shows again that the proposed ATGO and ATGO+ outperform the 10 competing methods using this new metric score.
>
> In addition, we list in Table S7 the ICW-Fmax scores performed by all 10 GO prediction methods, where ATGO/ATGO+ show again a better performance than other competing methods in all three GO aspects.

8. <u>The Reviewer commented:</u>

> ***2) I like the visualization of the different predictions showed in Fig 6.***

We appreciate the positive comments from the Reviewer.
